# Subtyping Alzheimer’s disease and Parkinson’s disease using longitudinal electronic health records

**DOI:** 10.1038/s43587-026-01085-3

**Published:** 2026-02-26

**Authors:** Jie Lian, Zhengxian Fan, Ben Omega Petrazzini, Wei Fan, Shishir Rao, Qianqian Yang, Guyu Zeng, Nouman Ahmed, Fatemeh Tabassi Mofrad, Malgorzata Wamil, Kazem Rahimi

**Affiliations:** 1 Deep Medicine, Nuffield Department of Women’s & Reproductive Health, University of Oxford, Oxford, UK; 2 Department of Psychiatry, Warneford Hospital, University of Oxford, Oxford, UK

**Keywords:** Neurodegenerative diseases, Predictive markers, Experimental models of disease, Ageing

## Abstract

Neurodegenerative diseases such as Alzheimer’s disease (AD) and Parkinson’s disease (PD) are clinically heterogeneous, hampering the success of nonselective treatment strategies. Here we apply a transformer-based unsupervised clustering framework to longitudinal electronic health record data from over 100,000 patients across two UK cohorts, Clinical Practice Research Datalink Aurum and UK Biobank, to identify, validate and characterize subtypes of AD and PD. We uncover five reproducible subtypes for each condition, characterized by distinct comorbidity patterns, symptom trajectories, outcomes and genetic profiles. These include a high-mortality AD subtype with motor and cardiovascular features, and a genetically susceptible but clinically resilient PD subtype. We also identify metabolic–inflammatory and vascular–psychiatric phenotypes shared across AD and PD, suggesting cross-disease mechanisms. By integrating routinely collected electronic health record data with genetic analyses, our study provides a scalable framework for early, biologically informed subtyping, laying the groundwork for future targeted interventions in neurodegenerative diseases.

## Abstract (web summary)

Using large-scale UK health records and machine learning, Lian and colleagues identify subtypes of Alzheimer’s and Parkinson’s disease, revealing shared clinical patterns and genetic differences that could support earlier diagnosis and more targeted care.

## Main

### Background

Neurodegenerative diseases (NDDs), such as Alzheimer’s disease (AD) and Parkinson’s disease (PD), represent a complex and growing public health challenge^1^. AD is quickly becoming one of the most disabling and costly diseases of the twenty-first century^2^, while PD is the second most common NDD, affecting roughly 2–3% of adults over the age of 65 years^3,4^. In 2021, over 3 billion people were living with a neurodegenerative condition worldwide, accounting for 443 million years of healthy life lost due to illness, disability and premature death^5^. Aging stands out as the primary nonmodifiable risk factor for most NDDs^6^. With population aging, the burden of NDDs is expected to increase. Hence, effective preventive strategies and a deeper understanding of these diseases are urgently needed.

Although AD and PD often co-occur in aging populations and share several risk factors, they have distinct clinical manifestations^2–4^. AD is the leading cause of dementia, typically characterized by the onset of memory loss and progressive cognitive decline. By contrast, PD primarily manifests with motor symptoms, such as tremor and rigidity; cognitive impairment in PD usually arises later in the disease course. Both diseases are marked by considerable heterogeneity in clinical presentation and disease trajectories^7–9^. This heterogeneity complicates diagnosis, prognosis and the development of therapeutics, frequently contributing to the failure of disease-modifying interventions^10–12^.

Consequently, there is growing research focused on subtyping AD and PD into more homogeneous groups to improve prognostic accuracy and accelerate the discovery of tailored therapies^13–24^. Subtyping efforts in AD and PD have utilized a wide range of approaches. For instance, in AD, subtypes have been proposed based on the distribution of tau neurofibrillary tangles^14^, neuroimaging^15^ and multimodal data^17^. Similarly, PD phenotypes have been proposed using genetic risk profiles, motor and nonmotor symptomatology and imaging^20–23^. Despite these advances, current subtyping research in AD and PD faces several key limitations. First, these approaches often suffer from small or selective cohorts and limited external validation, which challenge the generalizability of their findings^25–27^. Second, most subtyping research focuses primarily on disease progression, emphasizing features observable after disease onset^26,27^. However, prediagnostic information is often underutilized, limiting the understanding of potential disease causes and early risk factors and the clinical utility of the identified subtype. Furthermore, most existing subtyping studies in this field have relied on either clinical or genetic data in isolation, limiting phenotype-to-genotype interpretability.

Recent advances in large-scale electronic health records (EHRs) have opened new opportunities for subtyping complex disorders. EHR databases, such as the Clinical Practice Research Datalink (CPRD), capture rich longitudinal medical histories of millions of patients, enabling robust analyses of disease trajectories. In parallel, machine learning techniques have proven successful in identifying meaningful subtypes for conditions such as heart failure and diabetes, offering new insights into underlying biology and differing prognoses^28–33^. Yet, these approaches have rarely been applied to common NDDs such as AD and PD. Investigating AD and PD in parallel may reveal convergent patterns or shared risk mechanisms, offering broader insights into neurodegenerative processes. Moreover, integrative approaches that combine routinely collected clinical data with genetic variation may offer solutions for advancing precision medicine in NDDs.

In this study, we applied a three-stage framework to characterize phenotypic heterogeneity in AD and PD. First, we applied transformer-based deep learning models to EHR data to identify and validate subtypes on the basis of prediagnostic clinical information. Second, we analyzed the prognostic relevance of these subtypes in both internal and external datasets, describing differences in clinical outcomes and disease trajectories. Third, we investigated the association of these subtypes with genotype data, uncovering potentially different genetic underpinnings. By integrating routine clinical insights with genetic explanations, our approach aims to provide a scalable strategy for data-driven subtyping of NDDs and lay the groundwork for personalized disease modeling.

## Results

### Study design and patient characteristics

**Fig. 1:**
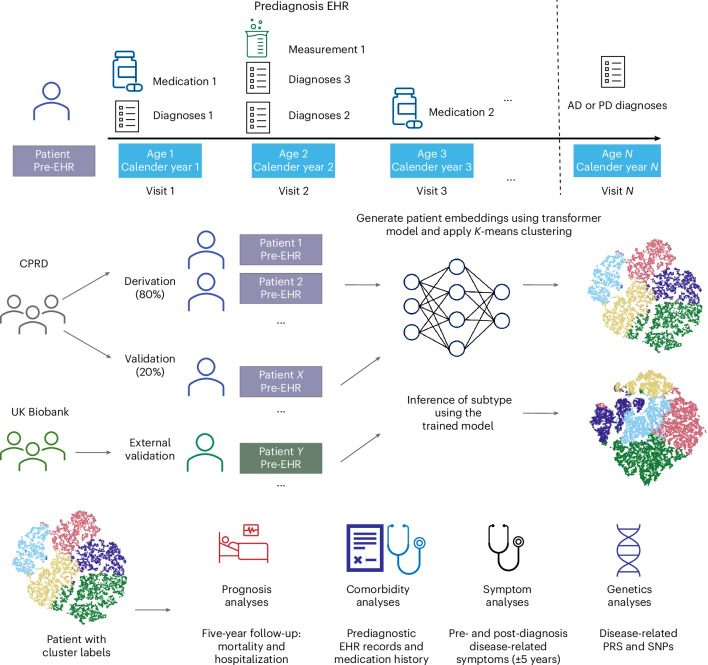
Study design and analytical workflow. Longitudinal prediagnostic EHRs from the CPRD and UK Biobank were used to subtype AD and PD. Each patient’s time-stamped EHR were tokenized by visit, age and calendar year to construct sequential inputs for a transformer model. Patient embeddings derived from the model were clustered using *K*-means to identify data-driven subtypes. The CPRD cohort was split into derivation (80%) and validation (20%) sets based on GP identifiers. UK Biobank served as an external validation dataset. Subsequent analyses compared clusters with respect to prognosis (5-year follow-up for mortality and hospitalization), comorbidities (prediagnostic EHR records and medication history), symptoms (pre- and post-diagnosis disease-related symptoms within ±5 years) and genetics (disease-related PRS and SNPs).

This study utilized CPRD Aurum^34^ as the primary data source, with UK Biobank^35^ serving as an external validation set. CPRD Aurum comprises extensive EHR data from UK general practices (GPs), covering approximately 20% of the UK population. The dataset includes patient demographics, clinical diagnoses, prescriptions, test results and lifestyle factors. Furthermore, CPRD is linked to Hospital Episode Statistics (HES) for secondary care data and the Office for National Statistics for mortality records, providing a comprehensive, de-identified dataset of a broad UK population. Refer to Fig. 1 and the Methods for details.

A total of 228,637 and 4,623 AD cases were identified in CPRD and UK Biobank, respectively, of which 113,545 and 3,710 patients met our inclusion criteria. For PD, CPRD and UK Biobank contained 95,408 and 4,685 cases, respectively, of which 45,825 and 3,732 patients were ultimately selected (see selection diagrams in Supplementary Figs. 1–4).

The AD cohort’s mean ages at diagnosis were 82.1 years (standard deviation (s.d.) 8.0) in CPRD and 74.4 years (s.d. 5.5) in UK Biobank. Females accounted for 63.8% of the CPRD cohort and 52.1% of the UK Biobank cohort. The cohorts were predominantly white (93.6% CPRD, 91.3% UK Biobank), with 22.3% (CPRD) and 34.5% (UK Biobank) classified as Index of Multiple Deprivation^36^ (IMD) category 1 (most deprived areas). Notably, 0.2% of patients with AD in CPRD and 0.1% in UK Biobank were aged between 40 and 50 years. In addition, 64.9% of CPRD patients were over 80 years old, compared with only 14.9% in UK Biobank.

In the PD cohorts, the mean ages at diagnosis were 77.8 years (s.d. 9.3) in CPRD and 70.6 years (s.d. 7.2) in UK Biobank, with females constituting 40.6% and 37.1% of the CPRD and UK Biobank cohorts, respectively. White individuals represented 93.3% of the CPRD and 90.7% of the UK Biobank cohorts. IMD category 1 was reported for 23.78% of CPRD and 37.88% of UK Biobank participants. Among PD patients aged 40–50 years, 0.93% were recorded in CPRD and 0.96% in UK Biobank. In addition, 45.11% of CPRD patients were older than 80 years, in contrast to only 5.84% in the UK Biobank cohort (Supplementary Table 1).

### Model validation and clustering stability

We used each patient’s prediagnostic EHR as input in this study. Patients contributed long prediagnostic observation periods, with median (interquartile range (IQR)) durations of 18.9 (7.9–31.1) years for AD and 19.1 (9.1–30.6) years for PD in CPRD, and 35.0 (21.0–53.0) years for AD and 30.0 (19.0–49.0) years for PD in UK Biobank (Supplementary Tables 2 and 3).

To cluster patients into different subtypes, we first transformed EHR data into vector representations using a transformer-based model^33^. Subsequently, we performed *K*-means clustering on the generated hidden representations through prediction strength analysis^37^ (Methods). Using the prediction strength threshold of 0.95, we identified five clusters each for AD and PD.

**Extended Data Fig. 1:**
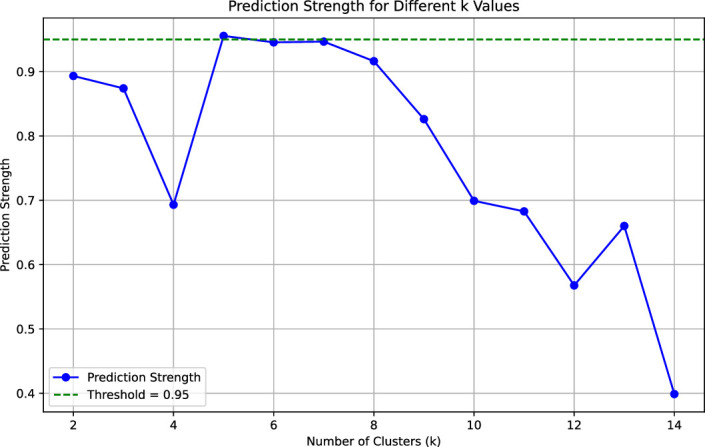
Prediction strength analysis for Alzheimer’s disease in the Clinical Practice Research Datalink validation dataset. Prediction strength values are shown for different numbers of clusters (k). A pre-specified threshold of 0.95 was used, and the largest value of k meeting this criterion was selected.

**Extended Data Fig. 2:**
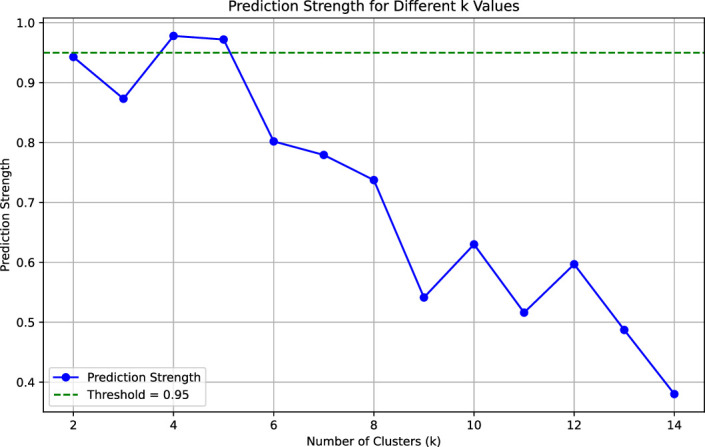
Prediction strength analysis for Parkinson’s disease in the Clinical Practice Research Datalink validation dataset. Prediction strength values are shown for different numbers of clusters (k). A pre-specified threshold of 0.95 was used, and the largest value of k meeting this criterion was selected.

**Extended Data Fig. 3:**
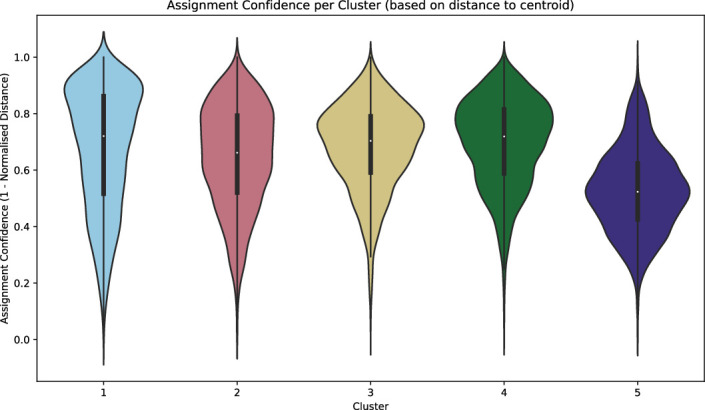
Alzheimer’s disease (AD) Assignment Confidence Distributions (N = 22,664). Violin plots show the distribution of assignment confidence for each AD cluster. Each violin contains a narrow box plot indicating the median (centre line), upper and lower quartiles (box limits), and whiskers extending to ±1.5 × the interquartile range (IQR); individual points represent outliers beyond the whiskers.

**Extended Data Fig. 4:**
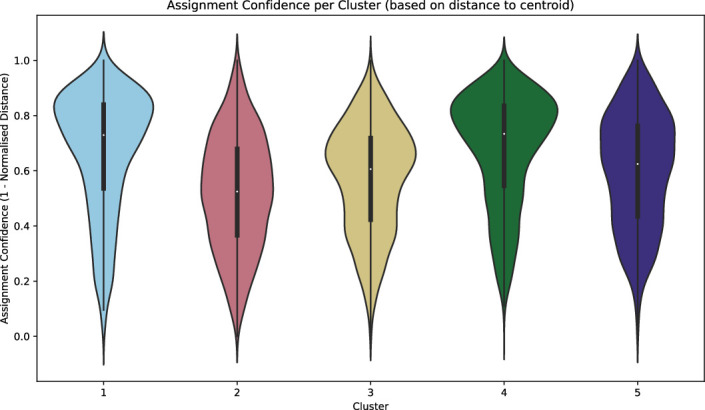
Parkinson’s disease (PD) Assignment Confidence Distributions (N = 8,946). Violin plots show the distribution of assignment confidence for each PD cluster. Each violin contains a narrow box plot indicating the median (centre line), upper and lower quartiles (box limits), and whiskers extending to ±1.5 × the interquartile range (IQR); individual points represent outliers beyond the whiskers.

The *t*-distributed stochastic neighbor embedding plots demonstrated clear cluster separation across the derivation, internal validation and UK Biobank datasets (Supplementary Figs. 5 and 6), with corresponding prediction strength plots in Extended Data Figs. 1 and 2. Assignment confidence distributions (Methods) further supported subtype cohesion, with most patients showing high alignment to their assigned cluster (Extended Data Figs. 3 and 4 and Supplementary Tables 4 and 5). In addition, we reported the other commonly used clustering metrics in Supplementary Tables 6 and 7, which consistently supported the robustness and validity of the identified clusters.

To benchmark performance, we compared our transformer-derived embeddings against two baseline representations: (1) term frequency–inverse document frequency with *K*-means clustering, and (2) a clinical-variable baseline including age, sex, IMD, calendar year, visit frequency and Charlson Comorbidity Index (CCI). The best-performing clusters from each baseline model, compared with our transformer-based approach, are summarized in Supplementary Table 8, demonstrating our model’s substantially superior clustering stability and reproducibility across all evaluation metrics.

### Five subtypes of AD and PD

**Fig. 2:**
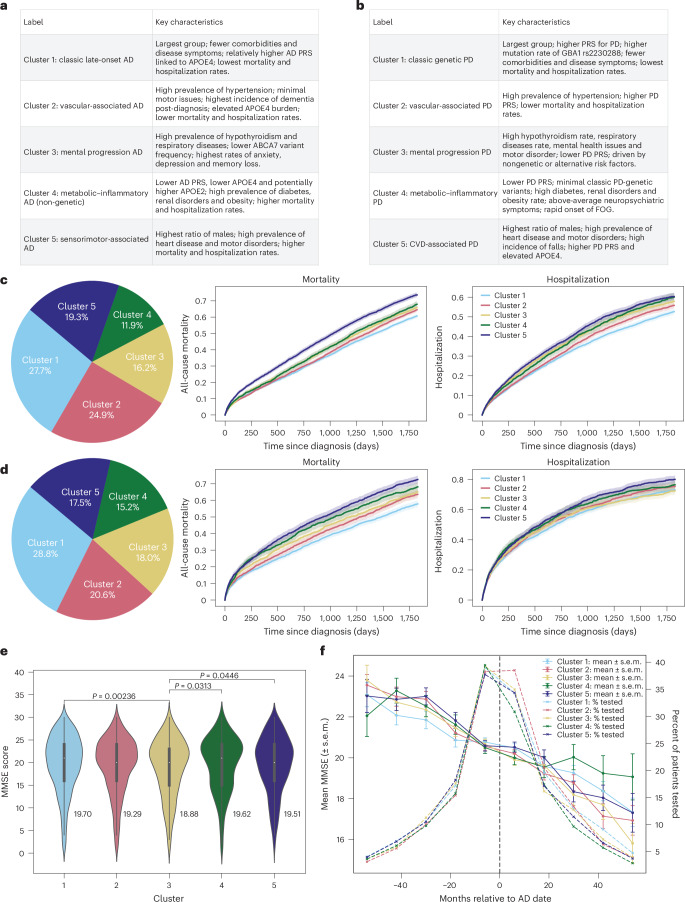
Cluster characteristics and prognostic outcomes for AD and PD. **a**,**b**, Cluster labels and main characteristics for both AD (**a**) and PD (**b**). **c**, AD population distribution on CPRD validation dataset (total *n* = 22,664), 5-year mortality and hospitalization rates for AD, mortality global log-rank *P* = 2.7 × 10^−50^; hospitalization global log-rank *P* = 3.0 × 10^−18^. **d**, PD population distribution (total *N* = 8,946), mortality global log-rank *P* = 2.1 × 10^−24^; hospitalization global log-rank *P* = 1.5 × 10^−4^. Solid lines represent the estimated survival or hospitalization rates, and shaded regions represent the 95% CIs (**c** and **d**). **e**, AD 5-year post-diagnosis mean MMSE scores across clusters. Data are shown as violin plots, with a narrow box-and-whisker overlay indicating the median (center line), upper and lower quartiles (box limits) and whiskers extending to ±1.5× the IQR; individual points beyond the whiskers represent outliers. Statistical differences between clusters were assessed using two-sided Mann–Whitney *U* tests. Number of samples: cluster 1, 1,618; cluster 2, 1,435; cluster 3, 863; cluster 4, 545, cluster 5, 989. **f**, The 10-year MMSE scores trend; the bars represent mean ± standard error of mean (s.e.m.) at each point. The dotted line represents AD diagnoses. Sample size per cluster (patients with more than one MMSE in 10 years): cluster 1, 1,767; cluster 2, 1,623; cluster 3, 1,046; cluster 4, 768, cluster 5, 1,179. Source data

We assigned descriptive labels to each subtype based on their predominant clinical and genetic features (see Fig. 2a–d for the cluster distribution). Cluster-wise baseline characteristics in the CPRD validation set are summarized in Table 1, and for the UK Biobank cohort in Supplementary Tables 9 and 10.

**Table 1:** Baseline characteristics by subtype of incident AD and PD in CPRD validation set (AD, *N* = 22,664; PD, *N* = 8,946)

	CPRD (AD)					CPRD (PD)				
	Cluster 1	Cluster 2	Cluster 3	Cluster 4	Cluster 5	Cluster 1	Cluster 2	Cluster 3	Cluster 4	Cluster 5
Age (years)	80.4 (9.1)	83.6 (7.2)	81.9 (8.0)	81.0 (7.2)	83.4 (6.9)	75.5 (10.3)	79.2 (8.4)	77.2 (9.7)	77.5 (8.5)	80.9 (7.4)
SBP (mm Hg)	135.0 (14.7)	140.9 (13.3)	135.8 (13.9)	135.1 (12.2)	133.7 (13.6)	132.0 (14.9)	139.9 (13.8)	133.7 (14.0)	135.1 (12.6)	132.2 (13.8)
BMI (kg m^−^^2^)	23.9 (4.4)	25.1 (4.7)	25.2 (5.1)	27.4 (5.0)	25.5 (4.4)	24.7 (4.5)	26.3 (4.6)	26.4 (5.4)	28.2 (5.2)	26.0 (4.3)
Male	2,272 (36.1%)	1,339 (23.8%)	863 (23.5%)	1,206 (44.7%)	2,568 (58.8%)	1,559 (60.6%)	1,027 (55.8%)	777 (48.4%)	923 (67.7%)	1,112 (71.2%)
Female	4,014 (63.9%)	4,295 (76.2%)	2,812 (76.5%)	1,495 (55.3%)	1,800 (41.2%)	1,014 (39.4%)	814 (44.2%)	829 (51.6%)	441 (32.3%)	450 (28.8%)
BMI obesity (BMI ≥27.5)	249 (4.0%)	464 (8.2%)	396 (10.8%)	650 (24.1%)	425 (9.7%)	142 (5.5%)	219 (11.9%)	256 (15.9%)	422 (30.9%)	172 (11.0%)
Smoker	381 (6.1%)	266 (4.7%)	312 (8.5%)	188 (7.0%)	286 (6.5%)	103 (4.0%)	68 (3.7%)	170 (10.6%)	91 (6.7%)	79 (5.1%)
Ex-smoker	523 (8.3%)	580 (10.3%)	555 (15.1%)	434 (16.1%)	678 (15.5%)	250 (9.7%)	218 (11.8%)	271 (16.9%)	256 (18.8%)	242 (15.5%)
Ethnicity white	5,770 (91.8%)	5,181 (92.0%)	3,448 (93.8%)	2,285 (84.6%)	4,142 (94.8%)	2,401 (93.3%)	1,704 (92.6%)	1,519 (94.6%)	1,151 (84.4%)	1,492 (95.5%)

For age, systolic blood pressure (SBP) and body mass index (BMI), we report the mean and s.d., while for the other features we report the number and percentage. Missing rate for AD: BMI 37.4%, SBP 4.6%. Missing rate for PD: BMI 36.5%, SBP 5.1%. Baseline variables (for example, BMI and SBP) were extracted as the latest available measurements within 2 years preceding the diagnosis date, following standard CPRD practice.

For AD, the clusters represented subtypes such as classic late-onset presentation (cluster 1), vascular-related patterns (cluster 2), neuropsychiatric dominance (cluster 3), metabolic–inflammatory profiles (cluster 4) and sensorimotor pattern (cluster 5). For PD, the clusters included classic genetic PD (cluster 1), vascular-associated types (cluster 2), severe neuropsychiatric forms (cluster 3), metabolic–inflammatory phenotypes (cluster 4) and cardiovascular–motor subtypes (cluster 5). We further identified the top 1% of patients closest to each cluster centroid (prototype patients), representing the most typical individuals, and summarized their demographic profiles and the most frequent clinical features (Supplementary Tables 11 and 12) to provide concrete clinical snapshots of each subtype.

Among the five AD subtypes, cluster 1 was the largest, comprising 27.7% of CPRD patients and 37.7% of UK Biobank patients. Clusters 1–3 were predominantly female, whereas cluster 5 included more male patients (58.8% in CPRD and 71.4% in UK Biobank). Cluster 4 showed a more balanced sex distribution (55.3% female in CPRD and 46.2% in UK Biobank). Age differences across AD clusters were relatively small: the largest mean age difference was 3.1 years in CPRD (cluster 1 versus cluster 2) and 2.2 years in UK Biobank (cluster 1 versus cluster 5). Notably, clusters 1 and 3 included the highest proportion of patients under 60 years of age.

For PD, cluster 1 was the most prevalent, representing 28.8% of CPRD and 40.0% of UK Biobank patients. Clusters 1, 2, 4 and 5 were predominantly male, while cluster 3 showed a balanced sex ratio (51.6% female in CPRD; 51.4% in UK Biobank). Age differences across PD clusters were modest: the largest mean age difference was 5.4 years in CPRD and 5.0 years in UK Biobank (both between cluster 1 and cluster 5). Only clusters 1 and 3 included patients under the age of 50. The detailed age distribution can be found in Supplementary Figs. 7–10.

Clusters (both AD and PD) differed in prediagnostic visit frequency and EHR density (all Kruskal–Wallis *P* < 0.0001), reflecting expected variation in healthcare utilization and disease complexity. However, GP-level effects were minimal (intraclass correlation coefficient 0.012 for PD and 0.008 for AD), indicating that <1% of cluster variance was attributable to GP practice (Supplementary Tables 13 and 14).

### Mortality and hospitalization

We observed differential mortality and hospitalization rates across the five identified subtypes (Fig. 2c). For patients with AD, cluster 1 had the lowest mortality and hospitalization rates, approximately 55% 5-year mortality and 50% hospitalization. This was followed by cluster 2, which comprised the oldest group in CPRD (mean age 83.6). Cluster 5 had the highest 5-year mortality and hospitalization rates. Mapping the clusters to the UK Biobank data revealed similar outcomes ranking among patient groups (Supplementary Fig. 11), with the exception that cluster 4 showed the highest mortality and hospitalization rates.

For patients with PD, cluster 1 similarly had the lowest mortality and hospitalization rates, approximately 50% for 5-year mortality and 65% for hospitalization. It was followed by cluster 2, which was the second-oldest group in CPRD (mean age 79.2). Cluster 5 recorded the highest 5-year mortality and hospitalization rates (Fig. 2d). Validation in UK Biobank data showed comparable trends (Supplementary Fig 12), although cluster 4 also had the highest mortality and hospitalization rates.

Kaplan–Meier curves showed significant differences in 5-year all-cause mortality and hospitalization across clusters (global log-rank *P* < 0.001; see Supplementary Tables 15–18 for pairwise results). Survival results remained consistent after multivariable adjustments for age, sex, IMD, calendar year, care intensity and recent comorbidity burden (2-year CCI), confirming that cluster–outcome associations were robust to demographic and healthcare use differences (Supplementary Tables 19 and 20). However, for PD, cluster membership was not significantly associated with hospitalization risk after adjustment.

### Subtype-specific comorbidities

**Fig. 3:**
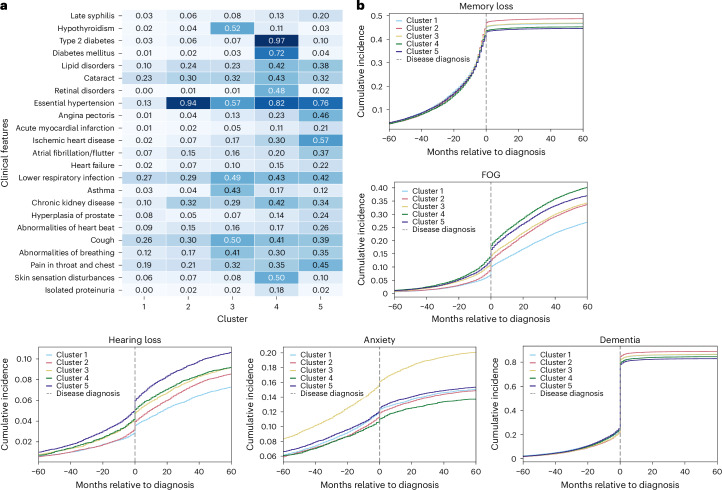
Comorbidities and disease-related symptoms for AD by subtype. **a**, Heatmap comorbidities for patients with AD, showing diseases with more than 15% variance across clusters in the CPRD validation dataset. Numbers in the heatmap represent the percentage of individuals within each cluster who have the corresponding diagnosis, normalized by the total number of individuals in that cluster. The color scale reflects the proportion (0–1) of individuals within each cluster with the corresponding diagnosis. **b**, Ten-year prevalence (5 years pre- and 5 years post-diagnosis) of symptoms for AD.

**Fig. 4:**
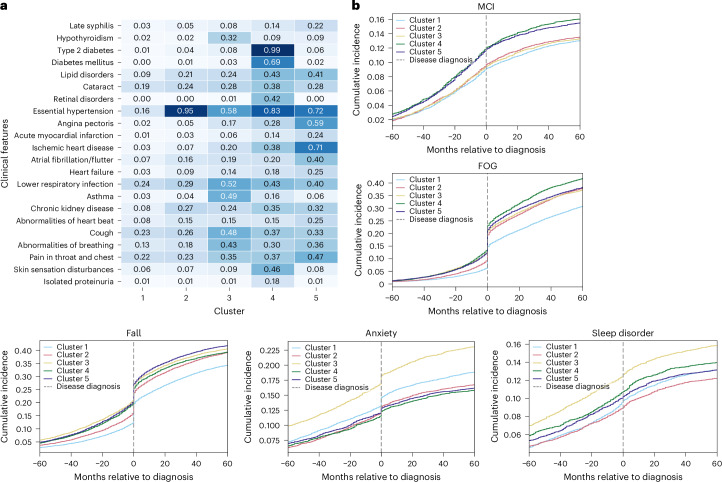
Comorbidities and disease-related symptoms for PD by subtype. **a**, Heatmap comorbidities for patients with PD, showing diseases with more than 15% variance across clusters in the CPRD validation dataset. Numbers in the heatmap represent the percentage of individuals within each cluster who have the corresponding diagnosis, normalized by the total number of individuals in that cluster. The color scale reflects the proportion (0–1) of individuals within each cluster with the corresponding diagnosis. **b**, Ten-year prevalence (5 years pre- and 5 years post-diagnosis) of symptoms for PD. MCI, mild cognitive impairment.

We summarized each subtype’s comorbidities to illustrate comorbidity heterogeneity (Fig. 3a for AD and Fig. 4a for PD). In addition to prevalence-based comparisons, we used weighted discriminative scores (WDS) to highlight codes that best distinguish each subtype. For each cluster, we visualized the top five discriminative diagnosis and medication codes using radar plots (Supplementary Figs 13 and 14).

For patients with AD, cluster 1 was characterized by low prevalence across all common diseases. Cluster 2 was predominantly defined by essential hypertension, affecting 94% of patients. Cluster 3 featured a high prevalence of hypothyroidism (52%) and respiratory diseases. Cluster 4 had notably high rates of diabetes (97%), renal disorders and skin sensation disturbances (50%), while cluster 5 was distinguished by cardiovascular diseases and a notable high 20% prevalence of late-stage syphilis. Validation with UK Biobank showed similar dominant comorbidities, except that skin sensation disturbances and late-stage syphilis were absent from clusters 4 and 5. Instead, musculoskeletal symptoms (32%) and gastrointestinal disorders (44%) emerged as secondary important comorbidities (Supplementary Fig 15). Radar plots were consistent with these dominant patterns: for example, essential hypertension, cognitive symptoms and diabetes-related medication codes were consistently top-ranked by WDS across cluster 2 and cluster 4, while cluster 3 stood out with high-ranking respiratory and thyroid-related features.

For patients with PD, the clustering revealed dominant predisease comorbidity profiles that closely mirrored those seen in the AD population. Within clusters, the dominant comorbidities were largely consistent between CPRD- and UK Biobank-derived profiles, with gastrointestinal disorders (52%) appearing as secondary important comorbidities in cluster 5 (Supplementary Fig 16). The radar plots are also consistent with these findings, highlighting distinct subtype signatures such as the hypertension-dominant profile in cluster 2 and the cardiovascular–motor pattern in cluster 5.

### Subtype-specific patterns of disease-related symptoms

To better understand early signals and disease progression, we examined 10-year trajectories of disease-related symptoms for each cluster, spanning from 5 years before to 5 years after disease onset. This highlighted early differences between disease subtypes and provided insights into how clinical symptoms evolve over time.

Among AD clusters, cluster 3 exhibited the highest incidence of depression and anxiety, highlighting a predominance of mental health problems. In addition, analyses of post-5-year mean Mini-Mental State Examination (MMSE) scores (Supplementary Tables 21 and 22) suggested that cluster 3 had notably lower performance, and the 10-year MMSE trend further revealed a slightly faster cognitive decline in cluster 3 compared with other clusters (Fig. 2e,f and Supplementary Fig 17). Clusters 4 and 5 experienced prominent motor disorders, including falls (Supplementary Fig 18), freezing of gait (FOG) and hearing loss. All clusters displayed high rates of memory loss and dementia (>80% dementia incidence post-diagnosis), with cluster 2 showing slightly higher and clusters 4 and 5 marginally lower prevalences (Fig. 3b).

In the PD cohorts, cluster 3 showed the most severe PD symptoms. It similarly demonstrated elevated anxiety and depression (Supplementary Fig. 19), alongside greater severity of motor-related symptoms, such as falls, FOG and sleep disorders, both before and after disease onset. In addition, cluster 3 patients exhibited earlier tremor symptoms (Supplementary Fig 20), up to 3 years before PD diagnosis. As for falls and FOG, cluster 3 initially had a higher presymptomatic prevalence, whereas cluster 4 experienced the most rapid progression of FOG post-diagnosis, followed closely by cluster 5. A similar pattern emerged for falls, with cluster 3 showing early signs and cluster 5 progressing more rapidly after diagnosis. In addition, clusters 4 and 5 displayed a higher prevalence of cognitive impairments (Fig. 4b), with cluster 5 also showing an increased prevalence of dementia (Supplementary Fig. 21).

### Genetic explanations of subtypes

To investigate the genetic underpinnings of the identified phenotypes, we conducted two types of comparison using disease-specific polygenic risk scores (PRS): pairwise comparisons between each cluster and all other clusters and the control group individually, and ‘1 versus others’ comparisons where each cluster was contrasted with all other clusters combined (for example, cluster 1 versus clusters 2–5). The control group consisted of Caucasian individuals without a diagnosis of AD or PD in the UK Biobank.

**Fig. 5:**
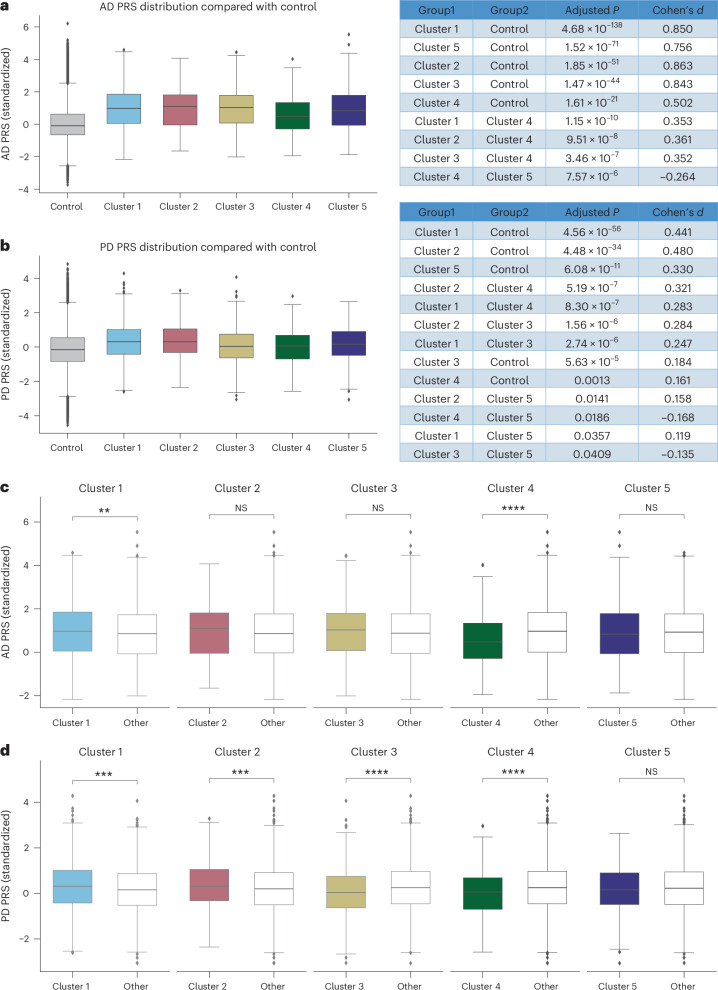
Genetic characteristics of AD and PD clusters. **a**,**b**, The distribution of AD (**a**) and PD (**b**) PRS across the five clusters relative to controls. Pairwise comparisons were performed using two-sided *t*-tests with Benjamini–Hochberg FDR correction; exact *P* values and effect sizes (Cohen’s *d*) are provided in the figures. **c**,**d**, ‘One-versus-all’ PRS comparisons for AD (**c**) and PD (**d**), where each cluster is contrasted with all other clusters combined using two-sided *t*-tests. *P* values for AD comparisons are as follows: cluster 1 (*P* = 0.003), cluster 2 (*P* = 0.109), cluster 3 (*P* = 0.149), cluster 4 (*P* = 2.14 × 10^−11^) and cluster 5 (*P* = 0.468). *P* values for PD comparisons are as follows: cluster 1 (*P* = 0.0001), cluster 2 (*P* = 0.0004), cluster 3 (*P* = 2.40 × 10^−5^), cluster 4 (*P* = 7.07 × 10^−6^) and cluster 5 (*P* = 0.390). For all box plots, the center line represents the median, box limits represent the upper and lower quartiles, and whiskers extend to 1.5× the IQR; points beyond whiskers indicate outliers. AD PRS analysis (**a** and **c**): control (*n* = 482,375), cluster 1 (*n* = 1,346), cluster 2 (*n* = 476), cluster 3 (*n* = 435), cluster 4 (*n* = 460), cluster 5 (*n* = 837); PD PRS analysis (**b** and **d**): control (*n* = 482,343), cluster 1 (*n* = 1,441), cluster 2 (*n* = 729), cluster 3 (*n* = 550), cluster 4 (*n* = 435), cluster 5 (*n* = 431). *P < 0.05, **P < 0.01, ***P < 0.001, ****P ≤ 0.0001. NS, not significant. Source data

As shown in Fig. 5a, for AD, all clusters exhibited significantly higher AD PRS scores than controls (mean PRS −0.01). Specifically, cluster 4 demonstrated a notably lower AD PRS score (mean 0.58) relative to each of the other clusters (cluster 1 mean: 0.99, *P* < 0.0001, cluster 2 mean: 0.99, *P* < 0.0001, cluster 3 mean: 0.99, *P* < 0.0001; cluster 5 mean: 0.89, *P* < 0.0001). Conversely, cluster 1 (Fig. 5c) showed a significantly higher PRS compared with the other cluster PRS (*P* = 0.0034). In addition, when comparing other conditions’ PRS scores across clusters (Supplementary Fig. 22), cluster 2 had elevated risks for hypertension and stroke; cluster 3 showed increased risks for asthma and rheumatoid arthritis; cluster 4 exhibited higher risks for type 1 and type 2 diabetes; and cluster 5 presented increased cardiovascular disease (CVD) risk.

For PD, all clusters displayed significantly higher PRS scores than controls (mean −0.12), although clusters 3 (mean 0.05) and 4 (mean 0.02) had significantly lower scores relative to the other clusters (Fig. 5b). Similar to AD, PRS scores for other diseases align with each cluster’s dominant comorbidities (Supplementary Fig 23). The ‘1 versus others’ analysis across PD clusters (Fig. 5d) revealed substantial variability in PD PRS, with clusters 1, 2, 3 and 4 each exhibiting significant differences.

To further explore subtype-specific genetic differences, we conducted two complementary single-nucleotide polymorphism (SNP) analyses. First, we performed additive logistic regression to model the association between minor-allele dosage (0, 1 and 2) and cluster membership (‘1 versus others’), adjusting for age, sex and the first three genetic principal components (GPC1–GPC3). Second, we conducted exploratory pairwise Fisher’s exact tests to directly compare carrier status between all cluster combinations. Because sample sizes varied across comparisons, no multiple-testing correction was applied for these descriptive contrasts.

In AD, additive logistic regression analyses (Supplementary Table 23) identified significant differences for *APOE4* (rs429358_C; cluster 4, odds ratio (OR) 0.62, 95% confidence interval (CI) 0.53–0.74, Bonferroni *P* = 1.9 × 10⁻⁶) and *APOE2* (rs7412_T; cluster 4, OR 1.80, 95% CI 1.30–2.49, Bonferroni *P* = 0.011), as well as *ABCA7* (rs3764650_G; cluster 1, OR 1.29, 95% CI 1.11–1.51, Bonferroni *P* = 0.032). Exploratory pairwise Fisher’s exact tests (Supplementary Table 24) corroborated these results, showing *APOE4* depletion and *APOE2* enrichment in cluster 4 (for example, cluster 4 versus cluster 5: OR 0.71, *P* = 0.004; cluster 4 versus 5 for *APOE2*: OR 1.38, *P* = 0.025) and *ABCA7* excess in cluster 1 versus 3 (OR 1.51, *P* = 0.005). Cluster-specific carrier enrichment (Supplementary Fig 24) further visualized these trends, with reduced *APOE4* (rs429358_C) and increased *APOE2* (rs7412_T) in cluster 4, consistent with a protective *APOE2* profile.

In PD, no variants remained significant after Bonferroni correction for regression analyses (Supplementary Table 25), although nominal associations were observed for *LRRK2* (rs34637584_A; cluster 2, OR 2.65, 95% CI 1.02–6.88, *P* = 0.046) and *APOE4* (rs429358_C; cluster 4, OR 0.79, 95% CI 0.64–0.98, *P* = 0.035). Pairwise comparisons (Supplementary Table 26) and carrier enrichment (Supplementary Fig. 25) indicated consistent patterns: *LRRK2* enrichment in cluster 2 and relative depletion of *APOE4* in cluster 4.

## Discussion

In this study, we identified five subtypes of AD and PD using large-scale longitudinal EHR data and a transformer-based framework. By leveraging prediagnostic clinical records, our approach captured phenotypic patterns that precede diagnosis of AD or PD, offering insights into early disease heterogeneity. Importantly, convergent clusters, such as metabolic–inflammatory and vascular–genetic phenotypes, across both diseases suggest shared clinical patterns of neurodegeneration.

Patients contributed long prediagnostic histories, allowing the model to learn from extended longitudinal patterns rather than short intervals. This design ensures that the identified subtypes reflect long-term clinical evolution and multimorbidity profiles rather than artifacts of diagnostic timing.

Previous EHR-based subtyping studies have provided valuable foundations for data-driven disease stratification^13,28,29,31,32,38^. Most, however, relied on hand-crafted, cross-sectional features and examined disease trajectories only after clinical onset. Other paradigms, such as consensus clustering and trajectory-based subtyping, have respectively focused on optimizing cluster stability across resampled datasets or explicitly modeling disease progression through predefined longitudinal features. By contrast, our framework applies deep representation learning to each patient’s complete, time-stamped prediagnostic trajectory, allowing temporal patterns to be captured rather than predefined. This approach identifies longitudinal subtypes that capture differences in clinical evolution and associated outcomes. While some overlap with earlier studies (for example, vascular or metabolic patterns) indicates convergent validity, our findings extend this work by providing a reproducible, data-driven description of clinical heterogeneity across AD and PD. Below, we outline key observations and their potential implications.

Although AD and PD are classified as distinct neurodegenerative syndromes, our clustering revealed subgroups characterized by vascular, metabolic and mental health comorbidities that appear across both diseases. These overlapping profiles may reflect shared systemic risk factors—such as vascular dysfunction, metabolic dysregulation or chronic inflammation—that influence disease expression or clinical course^28^. In particular, the co-occurrence of vascular and metabolic traits across AD and PD subgroups highlights how common systemic burdens may modulate neurodegenerative trajectories in the years preceding diagnosis^3^.

Evidence from prior literature supports this interpretation. Disruption of the blood–brain barrier, endothelial dysfunction and altered immune activation have been observed in both AD and PD, providing a plausible biological context for these shared profiles^39–42^. However, our findings are based on clinically recorded data and should be viewed as identifying correlated clinical patterns rather than proving mechanistic convergence. The reproducibility of these subtypes across independent cohorts and their distinct prognostic and genetic signatures nonetheless indicates that they capture genuine, disease-related heterogeneity rather than artifacts of data recording or general aging.

A recurring theme was the coexistence of high genetic susceptibility and vascular burden, particularly in cluster 2 for AD and PD, given that vascular dysfunction is a risk factor for neurological diseases^43^. In AD, this was characterized by extensive hypertension and elevated *APOE4* frequency, supporting the ‘mixed dementia’ construct, where vascular damage may potentiate amyloid toxicity^44,45^. In PD, patients with *LRRK2* mutations and hypertension has been reported to show comparatively milder progression, suggesting that vascular status could influence the phenotypic impact of genetic risk^46,47^. These vascular–genetic patterns highlight the potential importance of cardiovascular health as a modifier of neurodegenerative trajectories, even among genetically susceptible individuals.

Cluster 4 in both diseases exhibited a distinct metabolic–inflammatory signature, with high prevalence of pre-existing diabetes, renal disease and obesity. In particular, diabetes alters insulin signaling in the brain, which contributes to amyloid accumulation and neuronal degeneration^48^. Renal disease is linked with insufficient clearance of neurotoxic proteins, as observed in NDDs^49^, and obesity-related systemic inflammation plays a role in driving neurodegenerative processes^50^. Despite lower PRS, patients associated with cluster 4 experienced aggressive disease trajectories, including early symptom onset and high mortality. These findings add weight to the ‘type 3 diabetes’ hypothesis in AD and its analog in PD, supporting the notion that systemic metabolic dysregulation can mimic or exacerbate neurodegenerative processes^51–53^. Clinically, these observations highlight the importance of proactive metabolic screening and intervention in neurodegenerative risk management, especially where genetic risk is low.

A neuropsychiatric subtype (cluster 3) was observed in both conditions, defined by elevated depression and anxiety rates. These patients exhibited high symptom burden and faster cognitive decline in AD^54^, and tremor-dominant, nondemented phenotypes in PD^55,56^. These findings are consistent with prior evidence linking affective and stress-related disorders to altered neurodegenerative trajectories and may reflect the influence of systemic or nondopaminergic pathways, such as serotonergic or inflammatory processes. Although causal relationships cannot be inferred from EHR data, the prominence of mental health comorbidity highlights the potential importance of early neuropsychiatric management and integrated care in these populations.

Cluster 1 in both AD and PD showed high genetic predisposition (for example, high PRS) but relatively low comorbidity burden. This suggests the presence of protective modifiers—potentially related to vascular health, cognitive reserve or lifestyle factors. These resilient subtypes illustrate that genetic risk is not strongly deterministic for disease progression and offer opportunities to investigate protective pathways that may delay or prevent disease onset^57,58^.

Cluster 5 in both AD and PD was characterized by cardiovascular and motor system dysfunction, high hospitalization rates and poor survival, representing a severe multisystem phenotype. In AD, motor symptoms may suggest overlap with Lewy body or vascular dementia^59^; in PD, late-stage syphilis was also observed^60^. These clusters also showed elevated cardiovascular risk scores and, in PD, enrichment for the *APOE4* allele—a potentially important cross-disease association^61,62^. Collectively, these findings highlight the intersection between cardiovascular health and neurodegenerative progression and suggest that patients with substantial vascular and motor comorbidity may represent a clinically vulnerable subgroup warranting closer multidisciplinary monitoring in future studies.

While AD and PD maintain distinct core genetic risk profiles (for example, *APOE4*, *ABCA7* versus *GBA1*, *LRRK2*), systemic factors such as diabetes, depression and hypertension appeared to influence clinical trajectories across both diseases^18,24^. Of particular note, *APOE4* appeared in a subset of patients with PD with vascular burden, suggesting a potential lipid–inflammation link that may operate across NDDs^61,63^. Together, these observations point to the interplay between inherited susceptibility and modifiable systemic conditions and support future development of integrative risk frameworks that capture both genetic and comorbidity-related contributions to disease heterogeneity.

Our study has some limitations. First, although specialist hospital diagnoses in HES may in some cases reflect biomarker-confirmed assessments, such information is not systematically recorded in CPRD; therefore, we cannot check which patients had biomarker-confirmed diagnoses of AD or PD. Second, symptom and disease definitions relied on Systematized Nomenclature of Medicine—Clinical Terms (SNOMED-CT), Read, and International Statistical Classification of Diseases (ICD-10) codes, which may not capture all relevant symptoms or disease incidences owing to underrecording in GP or HES. For instance, key motor signs such as tremor may be underreported. Third, cognitive testing data were sparse—MMSE scores were available for only ~30% of participants—limiting granularity in cognitive phenotype mapping. Finally, as highlighted in recent work^64^, EHR-based studies of NDDs may be affected by detection bias linked to differential healthcare utilization. Although our models adjusted for visit frequency and comorbidity burden, and subtypes were independently replicated in the UK Biobank, residual detection bias cannot be entirely excluded.

Our EHR-based subtyping framework complements biology-defined approaches by capturing clinical heterogeneity observable in routinely collected healthcare data. The analysis is exploratory and hypothesis-generating, reflecting population-level variation rather than definitive biological mechanisms. Although we explored linking subtypes to brain imaging or cerebrospinal-fluid biomarkers in the UK Biobank, the number of cases with such data was too small for meaningful analysis. Nevertheless, the identified EHR-derived subtypes illustrate how routinely collected data can inform large-scale, noninvasive patient stratification. These findings highlight the potential for future multimodal integration as biomarker-linked datasets expand, helping to bridge routine clinical information with emerging biological models of NDDs.

Future studies should integrate more granular clinical and imaging data, standardized cognitive testing and temporally aligned longitudinal biomarker profiles. While our cohorts represent clinically diagnosed rather than biomarker-confirmed AD and PD, the identified subtypes demonstrated clear disease specificity through genetic and prognostic validation. Nonetheless, a future case–control design incorporating biomarker-defined AD and PD alongside nondementia comparators would be valuable to further validate the specificity of these clusters and clarify whether the vascular or metabolic profiles observed here are disease-specific or reflect broader aging-related processes.

## Methods

### Data

The CPRD cohort was stratified into a derivation cohort (80% of GPs) and an internal validation cohort (20% of GPs). This division ensured generalized model development and internal validation. UK Biobank, which has a similar EHR structure to CPRD, was used as an external validation dataset to assess model performance. In addition, UK Biobank provided genetic data, including PRS and SNPs, which were incorporated into the genetic characterization and explanatory analyses of each phenotype.

Access to UK Biobank data (application IDs 83942 and 116292) was obtained via UK Biobank’s standard access procedures. UK Biobank holds blanket ethical approval from the North West Multicentre Research Ethics Committee to function as a research tissue bank; therefore, investigators who work under an approved application are covered by that approval and do not require separate ethics clearance. The CPRD Aurum has ethical approval from a National Research Ethics Service committee for all purely observational studies. Additional study-specific approval for this analysis was granted by the CPRD Independent Scientific Advisory Committee (protocol 20_095). All UK Biobank participants provided written informed consent at enrollment. CPRD data are fully anonymized at the source and do not require individual patient consent.

### Cohort selection and case identification

We selected patients who were aged 40 years and older, with incident reports of AD or PD between 1 January 2005 and 1 January 2018. Selection criteria included adherence to CPRD quality standards, eligibility for CPRD and HES linkage, and a minimum of 12 months of registration with their GPs. The study period was restricted to 2005–2018 to maximize coding consistency and linkage completeness. Data quality in CPRD improved substantially after 2005 following the national implementation of SNOMED-CT and comprehensive HES linkage. The year 2018 was selected to ensure sufficient follow-up for post-diagnosis analyses.

AD and PD were identified on the basis of the first recorded diagnostic code from linked primary care (GP records) or secondary care (HES) data. The date of this first code was defined as the index date of diagnosis. In primary care, we extracted diagnoses using Read or SNOMED-CT code. In secondary care, we used ICD-10 codes. AD was identified using previously validated code lists^65^ (Supplementary Methods 1), while PD was identified using ICD-10 code of G20^66^. AD and PD cohorts were constructed separately. Patients carrying both codes were therefore included in both cohorts, as the AD and PD subtyping analyses were conducted independently.

### Data coverage and observation window

The CPRD Aurum and UK Biobank datasets are well-established and extensively validated UK national resources, covering approximately 20% and 6% of the population, respectively. Both link primary care (Read/SNOMED-CT), hospital episode (ICD-10; HES) and national mortality records, ensuring near-complete capture of longitudinal healthcare interactions. In the UK, all residents are registered with a GP, which serves as the central repository of clinical information and the anchor for secondary care linkage.

Patient medical records, encompassing diagnoses, medications, procedures and laboratory test results, were used as the input data. Only prediagnosis records were included to maximize the model’s clinical utility. A post-diagnosis observation window of 5 years was used to validate and interpret the clustering outcomes. Please refer to Fig. 1 for the overall study design.

To evaluate potential influences of healthcare utilization and practice-level recording variation, we compared visit frequency and record density across clusters using Kruskal–Wallis tests, and quantified practice-level variance using mixed-effects models with practice as a random intercept (intraclass correlation coefficient); detailed specifications are provided in Supplementary Methods 2.

### EHR representation learning

Longitudinal prediagnostic EHR data were transformed into patient-level vector representations using a transformer-based model^33^ trained on sequential clinical events. Diagnoses, procedures and medications were mapped to a unified clinical vocabulary and embedded jointly with temporal information, including patient age and calendar year at each encounter.

The model was first trained using a masked encounters modeling objective to capture latent co-occurrence patterns and temporal structure in EHR sequences. Patient representations were subsequently refined using contrastive learning to enhance disease-relevant separation. For each patient, two temporally contiguous segments of the prediagnostic record were sampled to form a positive pair, while sequences from different individuals were treated as negative pairs. Final patient embeddings were obtained by aggregating the first and final hidden layers of the transformer encoder, ensuring comprehensive EHR representation^67^. Detailed implementation and training procedures are provided in Supplementary Methods 3 and 4.

### Patient trajectory clustering and subtype selection

We then applied *K*-means clustering to the resulting patient embeddings, across a range of cluster numbers (*K* = 3 to 8). *K*-means was chosen for its scalability, simplicity and transparent centroid-based geometry, which are well suited to the continuous, approximately Euclidean structure of the transformer-derived EHR embeddings and facilitate clinical interpretation and cross-cohort reproducibility^68,69^. The optimal number of clusters was selected as the largest value of *K* achieving a prediction strength^37^ ≥0.95, which was prespecified as the primary criterion for evaluating out-of-sample reproducibility.

To further support this selection and strengthen interpretability, we assessed internal cohesion and separation using the silhouette score and Davies–Bouldin index; resampling stability using the bootstrap-adjusted Rand index (ARI); assignment consistency using the consensus clustering proportion of ambiguous clustering metric; and cross-cohort generalizability using cross-source ARI between CPRD and UK Biobank. We also applied *t*-distributed stochastic neighbor embedding plots to visualize the predicted clusters to illustrate patient subgroup separation and clustering quality. Full details of the clustering evaluation pipeline and selection rationale are provided in Supplementary Methods 5.

To assess assignment confidence and cluster cohesion under the nonprobabilistic *K*-means algorithm, we computed a distance-based confidence score for each patient as 1 minus the normalized Euclidean distance to their assigned cluster centroid. We then summarized these confidence scores using violin plots and descriptive statistics (mean, median, IQR, minimum and maximum) per cluster.

### Benchmarking with baseline models

To contextualize model performance, we developed two baseline approaches using identical patient cohorts and clustering evaluation procedures. The first used a term frequency–inverse document frequency representation of all prediagnostic coded events, followed by *K*-means clustering. The second used a baseline clinical variable, including age, sex, IMD, disease calendar year, mean annual visit frequency and 2-year CCI, as input features.

For each baseline, clustering was performed across *K* = 3–8, and the optimal configurations were selected based on (1) a prediction strength threshold of 0.95, consistent with the main model, and (2) the highest silhouette score. The two best-performing baseline models identified by these criteria were compared with our transformer-based model using the same internal and external validation metrics on the CPRD dataset.

### Prognostic analyses

After training the model and determining the optimal number of clusters in the derivation cohort, we applied the models to the validation cohort to identify and analyze patient clusters. First, we evaluated the prognostic value of the clustering approach by conducting survival analyses using the internal validation and UK Biobank. Kaplan–Meier cumulative incidence plots were used to illustrate 5-year all-cause mortality and disease-related hospitalization rates across clusters after diagnosis, with global and pairwise log-rank tests applied to assess statistical differences between curves.

To further assess robustness, we fit multivariable Cox proportional-hazards models adjusted for key demographic, socioeconomic and clinical covariates, including age, sex, IMD, calendar year, care intensity (mean annual visit frequency) and recent comorbidity burden quantified by the 2-year CCI.

### Comorbidity analyses

We assessed variance in the prevalence of recorded comorbidities and medications before the first diagnosis of AD and PD across clusters. Variables showing at least a 15% difference between the clusters with the highest and lowest prevalence were visualized using heatmaps to highlight potential pre-AD/PD comorbidity indicators. Cluster interpretation was further supported by the WDS, a metric that quantifies each code’s discriminative power across clusters by integrating both within-cluster prevalence and between-cluster separation (Supplementary Methods 6). For each cluster, we identified the top five most discriminative diagnoses and medications. These were visualized using per-cluster radar plots to provide interpretable subtype signatures.

In addition, we reported the prevalence of specific disease-related symptoms before and after 5 years to comprehensively characterize patient subtypes and explore disease progression and clinical heterogeneity, including dementia, falls, hearing loss, FOG, anxiety and depression for both AD and PD. For patients with AD, we also reported progression in MMSE scores and memory loss. For patients with PD, we evaluated tremor, MCI and sleep disturbances (see Supplementary Methods 7 and 8 for symptom definitions).

### Genetic analyses

To explore the genetic architecture of patient subtypes, we designed two sets of experiments using PRS and SNP on UK Biobank.

For the PRS analysis, we first compared the distribution of each cluster against every other cluster as well as the control group in terms of AD and PD PRS score. Then, we compared each cluster with the combined remainder of clusters to assess the uniqueness of their biological profiles relative to other diagnosed cases. Two-sided *t*-tests were implemented to explore the statistical difference. Pairwise cluster–cluster and cluster–control comparisons were tested using two-sided *t*-tests, and multiple testing correction was applied using the Benjamini–Hochberg false discovery rate (FDR). In addition to *P* values, we report effect sizes (Cohen’s *d*) to quantify the magnitude of differences. One-versus-rest comparisons were performed to evaluate the distinctness of each cluster relative to other diagnosed cases. In addition, we reported the mean values of 38 additional PRS for each cluster and visualized these results in a heatmap. Please refer to Supplementary Methods 9 for details. Data distribution was assumed to be normal, but this was not formally tested.

As for SNP analyses, we included SNPs associated with AD (*APOE*^62,70^, *TREM2*^71^ and *ABCA7*^71^) or PD (*APOE*^63^*, LRRK2*^72,73^, *PRKN*^73,74^ and *GBA1*^63,73^) in previous genetic association studies. SNP genotypes were encoded using additive dosage models (0, 1 and 2), representing minor-allele counts. For each disease, we fit logistic regression models comparing each cluster (‘1 versus others’) as the dependent variable, with SNP dosage as the predictor and adjusting for age, sex and the first three genetic principal components (GPC1–GPC3). Bonferroni correction was applied to control for multiple testing. Results are reported as ORs with 95% CIs and corrected *P* values. Furthermore, we calculated the carrier enrichment summarized in a heatmap, defined as the ratio of cluster-specific carrier prevalence to the general population minor allele frequency^75^. In addition, we performed exploratory pairwise two-sided Fisher’s exact tests to compare carrier status between all cluster combinations. These unadjusted tests provide descriptive validation of raw frequency differences given unequal sample sizes per cluster. Detailed calculation, quality control and data processing can be found in Supplementary Methods 10.

### Statistics and reproducibility

Sample sizes were determined by the availability of eligible participants in each cohort after applying predefined inclusion criteria, data linkage requirements and quality control procedures; no formal statistical power calculations were performed. This study was observational in nature, and data collection was not randomized. No blinding was applied during data collection or analysis. The assumptions of the statistical methods used were assessed where applicable. In particular, proportional hazards assumptions for Cox regression models were evaluated using standard diagnostic approaches. For clustering and representation learning analyses, robustness and reproducibility were assessed using prespecified prediction strength criteria, internal resampling-based validation and external validation across independent cohorts. No data points were excluded from the analyses beyond predefined cohort eligibility criteria, data quality control procedures and handling of missing data as described in the Methods. Consistency of key findings across datasets was used as the primary criterion for assessing reproducibility.

### Reporting summary

Further information on research design is available in the Nature Portfolio Reporting Summary linked to this article.

## Supplementary information

Supplementary Information Supplementary Methods 1–10 and Figs. 1–25.

Reporting Summary

Supplementary Tables Supplementary Tables 1–26.

## Source data

Source Data Fig. 2 Statistical source data.

Source Data Fig. 5 Statistical source data.

## Data Availability

Access to CPRD data, including UK primary care records and linked datasets such as HES, is subject to approval through CPRD’s Research Data Governance Process. These data cannot be shared directly by the authors. Qualified researchers may apply for access through the RDG application system (https://www.cprd.com/research-applications). The UK Biobank data used in this study were accessed under approved application numbers 83942 and 116292. UK Biobank data are available to researchers through a regulated application process (https://www.ukbiobank.ac.uk/), and cannot be publicly released by the authors.

## Code availability

All analyses were performed in Python (version 3.7+). Deep learning models were implemented using PyTorch (v1.8.1) together with the Hugging Face Transformers library (v4.10.2). Data preprocessing used pandas (v1.3.5), and clustering analyses (*K*-means, silhouette score, Davies–Bouldin index and ARI) used scikit-learn (v1.0.2). Visualizations were produced using matplotlib (v3.5.3) and seaborn (v0.12.2). Dimensionality reduction for cluster visualization was conducted using MulticoreTSNE (v0.1). Additional utilities included Grad-CAM (pytorch-gradcam, v0.2.1), torchvision (v0.9.1), torchaudio (v0.8.1) and torchdiffeq (v0.2.5). The code used for model training and subtyping is publicly available via GitHub at https://github.com/SereneLian/Subtyping_EHR_AD_PD.
