# Supplementary material for: Subtyping Alzheimer’s disease and Parkinson’s disease using longitudinal electronic health records
Source: Nat Aging. 2026 Feb 26;6(3):612–25. doi: 10.1038/s43587-026-01085-3 (PMC13004679; doi:10.1038/s43587-026-01085-3)

nature aging

Supplementary information https://doi.org/10.1038/s43587-026-01085-3

Subtyping Alzheimer's disease and Parkinson's disease using longitudinal electronic health records

In the format provided by the authors and unedited

Table of Contents

Supplementary Methods 2
Supplementary Method 1: Disease code for Alzheimer's disease (AD) and Parkinson's disease (PD). 2
Supplementary Method 2: Robustness and covariate-adjusted prognostic analyses........ 3
Supplementary Method 3: EHR data processing........ 4
Supplementary Method 4: Model training........ 5
Supplementary Method 5: Clustering evaluation and selection 7
Supplementary Method 6: Weighted discriminative scores 9
Supplementary Method 7: Alzheimer's disease (AD) and Parkinson's disease (PD) both used symptom code........ 10
Supplementary Method 8: Alzheimer's disease (AD) and Parkinson's disease (PD) specific symptom code definition 26
Supplementary Method 9: Polygenic risk scores (PRS) calculation........ 29
Supplementary Method 10: single-nucleotide polymorphism (SNP) extraction, quality control (QC) and visualisation........ 30

Supplementary Figures 32
Supplementary Figure 1 32
Supplementary Figure 2 33
Supplementary Figure 3 34
Supplementary Figure 4 35
Supplementary Figure 5 36
Supplementary Figure 6 37
Supplementary Figure 7 38
Supplementary Figure 8 38
Supplementary Figure 9 39
Supplementary Figure 10 39
Supplementary Figure 11 40
Supplementary Figure 12 40
Supplementary Figure 13 41
Supplementary Figure 14 42
Supplementary Figure 15 43
Supplementary Figure 16 43
Supplementary Figure 17 44
Supplementary Figure 18 45
Supplementary Figure 19 45
Supplementary Figure 20 46
Supplementary Figure 21 46
Supplementary Figure 22 47
Supplementary Figure 23 48
Supplementary Figure 24 49
Supplementary Figure 25 49

Supplementary Methods

Supplementary Method 1: Disease code for Alzheimer’s disease (AD) and Parkinson’s disease (PD).

AD		
Code	Code Type	Description
F00	ICD10	Dementia in Alzheimer's disease
F00.0	ICD10	Dementia in Alzheimer's disease with early onset
F00.1	ICD10	Dementia in Alzheimer's disease with late onset
F00.2	ICD10	Dementia in Alzheimer's disease, atypical or mixed type
F00.9	ICD10	Dementia in Alzheimer's disease, unspecified
G30	ICD10	Alzheimer’s disease
G30.0	ICD10	Alzheimer’s disease with early onset
G30.1	ICD10	Alzheimer’s disease with late onset
G30.8	ICD10	Other Alzheimer's disease
G30.9	ICD10	Alzheimer's disease unspecified
Eu00.	Read	[X] Dementia in Alzheimer's disease
Eu000	Read	[X]Dementia in Alzheimer's disease with early onset
Eu001	Read	[X]Dementia in Alzheimer's disease with late onset
Eu002	Read	[X]Dementia in Alzheimer's disease, atypical or mixed type
Eu00z	Read	[X]Dementia in Alzheimer's disease, unspecified
F110.	Read	Alzheimer’s disease
F1100	Read	Alzheimer’s disease with early onset
F1101	Read	Alzheimer’s disease with late onset
F112.	Read	Senile degeneration of brain
Fyu30	Read	[X] Other Alzheimer's disease
PD		
G20	ICD10	idiopathic Parkinson’s disease

Supplementary Method 2: Robustness and covariate-adjusted prognostic analyses

To confirm that the derived clusters were not explained by demographic or healthcare-use differences, and to assess the stability of prognostic associations, we performed a series of covariate-adjusted and practice-level sensitivity analyses.

Covariate-adjusted Cox models.
For each disease (AD and PD) and outcome (all-cause mortality and disease-related hospitalisation), we fitted multivariable Cox proportional-hazards models in the internal validation and UK Biobank cohorts. Cluster 1 served as the reference category. Models were adjusted for:

- **Age at diagnosis (years)**
- **Sex (male/female)**
- **Index of Multiple Deprivation (IMD)**, as a measure of socioeconomic status
- **Calendar year of diagnosis**, to capture temporal changes in coding or treatment
- **Care intensity**, defined as the mean annual visit frequency during the five years preceding diagnosis
- **Recent comorbidity burden**, quantified using the two-year Charlson Comorbidity Index (CCI)

Model estimates were expressed as hazard ratios (HRs) with 95 % confidence intervals. Adjusted HRs were compared with unadjusted results to evaluate whether cluster–outcome associations persisted after controlling for these covariates.

Practice-level and recording-intensity sensitivity analyses.
To examine whether clustering reflected healthcare utilisation or practice-specific recording patterns, we tested associations between cluster membership and (i) visit frequency, (ii) record count, and (iii) general-practice identifier. Continuous variables were compared across clusters using Kruskal–Wallis tests. Associations with practice were assessed by chi-square tests and quantified using a mixed-effects model with practice as a random intercept.
The intraclass correlation coefficient (ICC) was calculated as the proportion of variance in cluster assignment explained by practice-level differences:

$$\mathrm{ICC} = \frac{\sigma^2_{\mathrm{practice}}}{\sigma^2_{\mathrm{practice}} + \sigma^2_{\mathrm{residual}}}.$$

ICC values < 0.05 were interpreted as minimal site-level influence.

Supplementary Method 3: EHR data processing

The Clinical Practice Research Datalink (CPRD) integrates multiple coding systems for documenting medical data across primary care and hospital settings. Diagnoses are recorded using both Med codes and International Classification of Diseases, 10th Revision (ICD-10) codes, while medical procedures are classified under the OPCS-4 coding system. Medication prescriptions are encoded using the Dictionary of Medicines and Devices (dm+d) and the British National Formulary (BNF) codes.

To achieve a single analysis vocabulary, we mapped primary-care diagnosis codes (Med code) to ICD-10 categories at the three- or four-character level ("up to the fourth character"), e.g., G30.x for AD and G20 for PD. ("Fourth level" refers to the ICD-10 subcategory after the decimal, when present; some categories do not have a fourth character.) Similarly, dm+d codes for medications were mapped to their corresponding BNF section-level codes and Virtual Therapeutic Moiety (VTM) classifications, leveraging the NHS Digital SNOMED CT dictionary to enhance classification accuracy.

For the purpose of constructing a comprehensive longitudinal patient record, we compiled and integrated all mapped diagnostic, procedural, and prescription codes recorded prior to the first documented diagnosis of Alzheimer's disease (AD) and Parkinson's disease (PD). This dataset was further enriched with essential contextual information, including the patient's age at each recorded medical encounter and the calendar year in which each diagnosis, procedure, or prescription event occurred. By incorporating this level of detail, we ensured a robust representation of each patient's clinical trajectory, facilitating more precise analyses of disease progression and treatment patterns.

Supplementary Method 4: Model training

Pre-training phase

To enable the model to capture complex relationships within longitudinal electronic health records (EHRs), we first performed **unsupervised pre-training** on the full CPRD dataset using a *masked encounters modelling (MEM)* objective, adapted from BEHRT (Li et al., *Sci Rep*, 2020). In this phase, medical encounters within a patient's sequence were randomly masked, and the model was trained to predict the missing encounters from surrounding context. This approach allowed the transformer to learn latent co-occurrence patterns among diagnoses, medications, and procedures, as well as the broader temporal structure of patient trajectories.

Patient representation and contrastive learning

After pre-training, patient-level representations were constructed by averaging the model's first and final hidden layers, integrating both local and global contextual information. These representations were then refined using a contrastive learning objective to enhance disease-specific separation for Alzheimer's disease (AD) and Parkinson's disease (PD).

For each patient, a random cut point was selected within the pre-diagnostic EHR timeline (between GP registration and first diagnosis). Records before and after this point formed a positive pair, while sequences from different patients were treated as negative pairs. The model was trained with the Multiple Negatives Ranking Loss (InfoNCE objective) to minimise distances between temporally contiguous segments from the same patient while maximising distances to other patients. This process encourages embeddings to represent shared medical context while remaining robust to variability in record length and visit frequency.

Mitigating temporal bias

To prevent the model from relying solely on chronological cues (e.g., age or visit year) to distinguish disease stages, age and year tokens were masked during contrastive fine-tuning. This ensures that the learned embeddings reflect underlying clinical trajectories rather than explicit temporal markers, leading to more generalisable patient representations.

Hyperparameters and Training Configuration

• Batch size: 64

• Maximum sequence length: 250

• Learning rate: 3e-5

• Number of epochs: 10

• Evaluation frequency: Every 500 steps

• Early stopping: Triggered if the loss remains unchanged for five consecutive evaluations

• Loss function: Multiple Negative Ranking Loss

Tokenizer and vocabulary

The model used a custom CPRD-specific vocabulary derived from Read, SNOMED-CT, and BNF ontologies, containing approximately 90,000 unique clinical tokens. Each token corresponds to a diagnosis, procedure, or medication recorded during a healthcare encounter, with additional vocabularies for age (111 tokens) and calendar year (37 tokens, 1985–2021). Tokenisation followed the BEHRT framework (Li et al., Sci Rep, 2020), in which clinical and temporal tokens were embedded jointly to enable the model to learn both disease co-occurrence and temporal progression patterns.

Training dataset and embedding dimension

Pretraining was performed on the full CPRD EHR corpus (>30 million linked primary and secondary care patients). Contrastive fine-tuning and clustering were conducted exclusively on the CPRD derivation cohort, with validation (CPRD internal) and external testing (UK Biobank) remaining unseen during model training. The final embedding dimension, obtained by averaging the first and last hidden layers, was 728, balancing representational richness and computational efficiency for subsequent clustering analyses.

Supplementary Method 5: Clustering evaluation and selection

Our goal was to derive robust, transferable subtypes from real-world EHR. Because coding practices, and care pathways vary across settings, we prioritised out-of-sample stability and external generalisability over purely geometric criteria. Below we detail the clustering workflow, validation metrics and selection rule for k.

Embeddings and clustering

Embeddings. Patient representations were obtained from a transformer model fine-tuned with a contrastive objective on sequential EHR (Methods, Supplementary Method 2). For each patient we mean-pooled the token embeddings (average of first and last encoder layers) with attention masking to form a single vector.

Clustering. We applied k-means to the embeddings with n_init=10, max_iter=50, and fixed random seeds. We evaluated k from 3 to 8.

Primary selection criterion: Prediction Strength (PS)

We pre-specified PS as the primary metric for selecting the number of clusters. PS measures the out-of-sample reproducibility of cluster assignments under data splitting, and penalises over-partitioning—making it particularly suitable for noisy, high-dimensional EHR representations. Our selection rule was to choose the largest value of K for which PS ≥ 0.95. Both AD and PD satisfied this threshold at $K = 5$ (see Supplementary Figures S7–S8).

Additional metrics

- Internal structure
    - Silhouette score (higher = better)
    - Davies–Bouldin index (lower = better)

These indices assess compactness and separation based on Euclidean geometry, but do not account for reproducibility.

- Resampling-based stability
    - We computed bootstrap Adjusted Rand Index (ARI) across 10 replicates by randomly resampling the cohort, clustering twice per replicate, and computing ARI between clusterings. This reflects the robustness of clustering outcomes under perturbation.
- Consensus clustering
    - We ran K-means 100 times per K and calculated the Proportion of Ambiguous Clustering (PAC). Lower PAC indicates greater label stability across repeated runs.
- Cross-source reproducibility
    - Using the CPRD derivation centroids, we assigned cluster labels to CPRD validation and UK Biobank patients and computed cross-source ARI between those assigned labels and the derivation labels.

Clinical validity

Kaplan–Meier curves for all-cause mortality and hospitalisation showed significant separation across clusters.

External replication and transportability

We assessed label transfer from CPRD to UK Biobank (and vice versa) by assigning UKB patients to CPRD centroids.

Prediction strength calculation

PS is a stability-based index that quantifies how reproducibly a clustering solution generalises to unseen data. It is widely used to determine the optimal number of clusters when no ground truth labels exist. For a given number of clusters k, the dataset is randomly divided into two halves. Clustering is performed independently on each half, and the resulting partitions are compared by reassigning each observation from one split to the nearest centroid of the other.Formally, if $C_i^{(1)}$and $C_j^{(2)}$denote the cluster assignments in the two random halves of the dataset, the prediction strength (PS) for cluster $i$is defined as:

$$\mathrm{PS}(k) = \min_i \frac{1}{\mid C_i^{(1)} \mid (\mid C_i^{(1)} \mid -1)} \sum_{\substack{a \neq b \\ a,b \in C_i^{(1)}}} \mathbf{1}\,[\,\mathrm{reassign}(a) = \mathrm{reassign}(b)\,],$$

where $\mathbf{1}[\cdot]$is the indicator function that equals 1 if its argument is true and 0 otherwise.

A higher PS indicates greater stability and reproducibility of clusters. Following the original publication and subsequent biomedical applications (2-3), we adopted PS ≥ 0.95 as a conservative threshold for stability—meaning that at least 95% of pairwise cluster assignments were reproducible across random train–test partitions.

Supplementary Method 6: Weighted discriminative scores

Unsupervised subtypes require transparent descriptors of what makes one group distinct from the remainder. Simple prevalence contrasts highlight common codes but not specificity; fold-change or odds ratios capture enrichment but can over-emphasise extremely rare codes. We therefore used a presence-weighted information measure—the Weighted Discriminative Score (WDS)—to rank codes that are both enriched in a subtype and sufficiently prevalent to be informative.

Mathematically, WDS equals the in-cluster prevalence multiplied by the log-ratio of in- vs out-cluster prevalences. This corresponds to the 'presence' contribution of a Bernoulli Kullback–Leibler (KL) divergence, so the score can be read in bits of information: how much evidence the presence of a code provides in favour of a given subtype.

Let $x \in \{0,1\}^{n \times p}$ denote the one-hot matrix of codes and y_i the subtype label. For cluster k and code j, define the in-cluster and out-cluster empirical prevalences:

$$\hat{p}_{in(j,k)} = \left(\frac{1}{n_k}\right) \sum_{\{i:\, y_i = k\}} x_{\{ij\}}$$

$$\hat{p}_{out(j,k)} = \left(\frac{1}{n_k}\right) \sum_{\{i:\, y_i \neq k\}} x_{\{ij\}}$$

To avoid division by zero we use a small additive smoothing constant $\varepsilon = 1e{-}6$.The weighted WDS is defined as:

$$WDS_{\{j,k\}} = \hat{p}_{in(j,k)} * \log_2\left(\frac{\hat{p}_{in(j,k)} + \varepsilon}{\hat{p}_{out(j,k)} + \varepsilon}\right)$$

Supplementary Method 7: Alzheimer’s disease (AD) and Parkinson’s disease (PD) both used symptom code

Dementia

Code (SNOMEDCT)	Term
101421000119107	Dementia due to Parkinson's disease (disorder)
10349009	Multi-infarct dementia with delirium (disorder)
10532003	Primary degenerative dementia of the Alzheimer type, presenile onset, with depression (disorder)
105421000119105	Early onset Alzheimer's disease with behavioral disturbance (disorder)
106021000119105	Multi-infarct dementia due to atherosclerosis (disorder)
1089501000000100	Presenile dementia with psychosis (disorder)
1089521000000100	Predominantly cortical dementia (disorder)
1089531000000100	Predominantly cortical vascular dementia (disorder)
1089581000000100	Predominantly cortical dementia (disorder)
111480006	Psychoactive substance-induced organic dementia (disorder)
1156789004	Autosomal dominant Alzheimer disease due to mutation of amyloid precursor protein (disorder)
1156798001	Autosomal dominant Alzheimer disease due to mutation of presenilin 2 (disorder)
1156800008	Autosomal dominant Alzheimer disease due to mutation of presenilin 1 (disorder)
12348006	Presenile dementia (disorder)
12741002	AIDS with dementia (disorder)
130121000119104	Dementia due to Rett syndrome (disorder)
13092008	Pick's disease (disorder)
135811000119107	Lewy body dementia with behavioral disturbance (disorder)
138736007	H/O: dementia (situation)
14070001	Multi-infarct dementia with depression (disorder)
141991000119109	Delusions in Alzheimer's disease (disorder)
142001000119106	Depressed mood in Alzheimer's disease (disorder)
142011000119109	Alzheimer's disease co-occurrent with delirium (disorder)
142811000119104	Dementia due to Alzheimer's disease (disorder)
154845000	Dementia (& [presenile] or [senile]) (disorder)
154846004	Uncomplicated senile dementia (disorder)
154847008	Presenile dementia (disorder)
154848003	Senile dementia with depressive or paranoid features (disorder)
154849006	Senile dementia with delirium (disorder)
154850006	Vascular dementia (disorder)
154851005	Other senile/presenile dementia (disorder)

155006000	Huntington's disease (disorder)
15662003	Senile dementia (disorder)
1581000119101	Dementia of the Alzheimer type with behavioral disturbance (disorder)
1591000119103	Dementia with behavioral disturbance (disorder)
161465002	History of dementia (situation)
16276361000119100	Vascular dementia without behavioral disturbance (disorder)
1823871000006100	Dementia confirmed (situation)
191448002	Senile and presenile organic psychotic conditions (& dementia) (disorder)
191449005	Uncomplicated senile dementia (disorder)
191450005	Presenile dementia (disorder)
191451009	Uncomplicated presenile dementia (disorder)
191452002	Presenile dementia with delirium (disorder)
191454001	Presenile dementia with paranoia (disorder)
191455000	Presenile dementia with depression (disorder)
191457008	Senile dementia with depressive or paranoid features (disorder)
191458003	Senile dementia with paranoia (disorder)
191459006	Senile dementia with depression (disorder)
191460001	Senile dementia with depressive or paranoid features NOS (disorder)
191461002	Senile dementia with delirium (disorder)
191462009	Arteriosclerotic dementia (including [multi infarct dementia]) (disorder)
191463004	Uncomplicated arteriosclerotic dementia (disorder)
191464005	Arteriosclerotic dementia with delirium (disorder)
191465006	Arteriosclerotic dementia with paranoia (disorder)
191466007	Arteriosclerotic dementia with depression (disorder)
191467003	Arteriosclerotic dementia NOS (disorder)
191474008	Alcoholic dementia: [other] or [NOS] (disorder)
191493005	Dementia caused by drug (disorder)
191519005	Dementia associated with another disease (disorder)
192160005	[X]Dementia in Alzheimer's disease (disorder)
192161009	Dementia in Alzheimer's disease with early onset (disorder)
192162002	Dementia in Alzheimer's disease with late onset (disorder)
192163007	[X]Dementia in Alzheimer's dis, atypical or mixed type (disorder)
192164001	[X]Dementia in Alzheimer's disease, unspecified (disorder)
192165000	Vascular dementia (disorder)
192166004	Vascular dementia of acute onset (disorder)
192167008	[X]Dementia: [multi-infarct] or [predominantly cortical] (disorder)
192168003	Subcortical vascular dementia (disorder)
192169006	Mixed cortical and subcortical vascular dementia (disorder)

192170007	[X]Other vascular dementia (disorder)
192171006	[X]Vascular dementia, unspecified (disorder)
192173009	[X]Dementia in other diseases classified elsewhere (disorder)
192174003	[X]Dementia in Pick's disease (disorder)
192175002	[X]Dementia in Creutzfeldt-Jakob disease (disorder)
192176001	[X]Dementia in Huntington's disease (disorder)
192177005	[X]Dementia in Parkinson's disease (disorder)
192178000	Acquired immune deficiency syndrome dementia complex (disorder)
192803007	Dementia in Alzheimer's disease with late onset (disorder)
20484008	Prion disease (disorder)
21921000119103	Dementia co-occurrent and due to Pick's disease (disorder)
22381000119105	Primary degenerative dementia (disorder)
229672009	Language disorder of dementia (disorder)
230258005	Amyotrophic lateral sclerosis with dementia (disorder)
230265002	Familial Alzheimer's disease of early onset (disorder)
230266001	Non-familial Alzheimer's disease of early onset (disorder)
230267005	Familial Alzheimer's disease of late onset (disorder)
230268000	Non-familial Alzheimer's disease of late onset (disorder)
230269008	Focal Alzheimer's disease (disorder)
230270009	Frontotemporal dementia (disorder)
230271008	Pick's disease with Pick bodies (disorder)
230272001	Pick's disease with Pick cells and no Pick bodies (disorder)
230273006	Frontotemporal degeneration (disorder)
230274000	Frontal lobe degeneration with motor neurone disease (disorder)
230280008	Progressive aphasia in Alzheimer's disease (disorder)
230281007	Argyrophilic grain disease (disorder)
230282000	Post-traumatic dementia (disorder)
230283005	Punch drunk syndrome (disorder)
230284004	Spongiform encephalopathy (disorder)
230285003	Vascular dementia of acute onset (disorder)
230286002	Subcortical vascular dementia (disorder)
230287006	Mixed cortical and subcortical vascular dementia (disorder)
230288001	Semantic dementia (disorder)
230289009	Patchy dementia (disorder)
230290000	Epileptic dementia (disorder)
230299004	Juvenile onset Huntington's disease (disorder)
230300007	Late onset Huntington's disease (disorder)
230301006	Akinetic-rigid form of Huntington's disease (disorder)
231463001	Alcoholic dementia NOS (disorder)
2421000119107	Hallucinations co-occurrent and due to late onset dementia (disorder)

25772007	Multi-infarct dementia with delusions (disorder)
26852004	Primary degenerative dementia of the Alzheimer type, senile onset, with depression (disorder)
268612007	Senile and presenile organic psychotic conditions (disorder)
268613002	Vascular dementia (disorder)
268615009	Other alcoholic dementia (disorder)
268675002	[X]Unspecified dementia (disorder)
268744003	Dementia (& [presenile] or [senile]) (disorder)
26929004	Alzheimer's disease (disorder)
278855005	Frontal lobe degeneration (disorder)
278857002	Dementia of frontal lobe type (disorder)
279982005	Cerebral degeneration presenting primarily with dementia (disorder)
281004	Dementia associated with alcoholism (disorder)
288631000119104	Vascular dementia with behavioral disturbance (disorder)
304603007	Variant Creutzfeldt-Jakob disease (disorder)
31081000119101	Presenile dementia with delusions (disorder)
312991009	Senile dementia of the Lewy body type (disorder)
32875003	Inhalant-induced persisting dementia (disorder)
371024007	Senile dementia with delusion (disorder)
371026009	Senile dementia with psychosis (disorder)
41161000000104	Multi-infarct dementia
414351000000102	[X]Dementia in Creutzfeldt-Jakob disease (disorder)
414361000000104	[X]Dementia in Huntington's disease (disorder)
416780008	Primary degenerative dementia of the Alzheimer type, presenile onset (disorder)
416975007	Primary degenerative dementia of the Alzheimer type, senile onset (disorder)
419261000000107	[X]Dementia in Alzheimer's dis, atypical or mixed type (disorder)
420614009	Organic dementia with acquired immunodeficiency syndrome (disorder)
421023003	Presenile dementia with acquired immunodeficiency syndrome (disorder)
421529006	Dementia with acquired immunodeficiency syndrome (disorder)
425390006	Dementia associated with Parkinson's Disease (disorder)
42769004	Diffuse Lewy body disease with spongiform cortical change (disorder)
429458009	Dementia due to Creutzfeldt Jakob disease (disorder)
429998004	Vascular dementia (disorder)
432111000000104	[X] (Mental and behavioural disorders due to use of alcohol: residual and late-onset psychotic disorder) or (chronic alcoholic brain syndrome [& dementia NOS]) (disorder)
442344002	Dementia due to Huntington chorea (disorder)
443491000000105	[X]Dementia in Alzheimer's disease (disorder)

443541000000101	[X]Dementia in Alzheimer's disease, unspecified (disorder)
455381000000102	[X]Dementia in Pick's disease (disorder)
45864009	Senile degeneration of brain (disorder)
464291000000103	[X]Dementia in other diseases classified elsewhere (disorder)
471341000000106	[X]Unspecified dementia (disorder)
4817008	Primary degenerative dementia of the Alzheimer type, senile onset, with delirium (disorder)
51928006	General paresis - neurosyphilis (disorder)
52448006	Dementia (disorder)
54502004	Primary degenerative dementia of the Alzheimer type, presenile onset, with delusions (disorder)
55009008	Primary degenerative dementia of the Alzheimer type, senile onset, with delusions (disorder)
56267009	Multi-infarct dementia (disorder)
58756001	Huntington's chorea (disorder)
59651006	Sedative, hypnotic AND/OR anxiolytic-induced persisting dementia (disorder)
613601000000106	Senile dementia with depressive or paranoid features NOS (disorder)
62102009	AIDS with presenile dementia (disorder)
62239001	Parkinson-dementia complex of Guam (disorder)
6475002	Primary degenerative dementia of the Alzheimer type, presenile onset, uncomplicated (disorder)
65096006	Primary degenerative dementia of the Alzheimer type, presenile onset, with delirium (disorder)
66108005	Primary degenerative dementia of the Alzheimer type, senile onset, uncomplicated (disorder)
671511000000100	Alcoholic dementia NOS (disorder)
67155006	Gerstmann-Straussler-Scheinker syndrome (disorder)
698624003	Dementia associated with cerebral lipidosis (disorder)
698625002	Dementia associated with normal pressure hydrocephalus (disorder)
698626001	Dementia associated with multiple sclerosis (disorder)
698687007	Post-traumatic dementia with behavioral change (disorder)
698725008	Dementia associated with neurosyphilis (disorder)
698726009	Dementia associated with viral encephalitis (disorder)
698781002	Dementia associated with cerebral anoxia (disorder)
698948009	Vascular dementia in remission (disorder)
698949001	Dementia in remission (disorder)
698954005	Primary degenerative dementia of the Alzheimer type, senile onset in remission (disorder)
698955006	Primary degenerative dementia of the Alzheimer type, presenile onset in remission (disorder)
700911000000100	Other senile/presenile dementia (disorder)
702429008	Frontotemporal dementia with parkinsonism-17 (disorder)

70936005	Multi-infarct dementia, uncomplicated (disorder)
713060000	Sporadic Creutzfeldt-Jakob disease (disorder)
713488003	Presenile dementia co-occurrent with human immunodeficiency virus infection (disorder)
713844000	Dementia co-occurrent with human immunodeficiency virus infection (disorder)
715737004	Parkinsonism co-occurrent with dementia of Guadeloupe (disorder)
716667005	Right temporal atrophy variant frontotemporal dementia (disorder)
722600006	Non-amnestic Alzheimer disease (disorder)
722977005	Dementia co-occurrent and due to neurocysticercosis (disorder)
722978000	Toxic dementia (disorder)
722979008	Dementia due to metabolic abnormality (disorder)
722980006	Dementia due to chromosomal anomaly (disorder)
723123001	Ischemic vascular dementia (disorder)
723390000	Rapidly progressive dementia (disorder)
724776007	Dementia due to disorder of central nervous system (disorder)
724777003	Dementia due to infectious disease (disorder)
724992007	Epilepsy co-occurrent and due to dementia (disorder)
725898002	Delirium co-occurrent with dementia (disorder)
733184002	Dementia caused by heavy metal exposure (disorder)
733185001	Dementia following injury caused by exposure to ionizing radiation (disorder)
733190003	Dementia due to primary malignant neoplasm of brain (disorder)
733191004	Dementia due to chronic subdural hematoma (disorder)
733192006	Dementia due to herpes encephalitis (disorder)
733193001	Dementia co-occurrent and due to progressive multifocal leukoencephalopathy (disorder)
733194007	Dementia co-occurrent and due to Down syndrome (disorder)
762350007	Dementia due to prion disease (disorder)
762351006	Dementia due to and following injury of head (disorder)
762707000	Subcortical dementia (disorder)
783161005	Familial dementia British type (disorder)
783258000	Familial dementia Danish type (disorder)
78511000000104	Multi-infarct dementia
788861009	Aggression due to dementia (finding)
788862002	Agitation due to dementia (finding)
788866004	Anxiety due to dementia (finding)
788867008	Apathetic behavior due to dementia (finding)
788898005	Dementia caused by volatile inhalant (disorder)
789170003	Disinhibited behavior due to dementia (disorder)
792004	Jakob-Creutzfeldt disease (disorder)
79341000119107	Mixed dementia (disorder)

79358009	Transmissible virus dementia (disorder)
80098002	Diffuse Lewy body disease (disorder)
816461000000109	Suspected dementia (situation)
82351000119105	Altered behavior due to Pick's disease (disorder)
82361000119107	Altered behavior in dementia due to Huntington chorea (disorder)
82371000119101	Dementia due to multiple sclerosis with altered behavior (disorder)
82381000119103	Epileptic dementia with behavioral disturbance (disorder)
82959004	Dementia paralytica juvenilis (disorder)
83157008	Fatal familial insomnia (disorder)
833326008	Cortical vascular dementia (disorder)
838276009	Amyotrophic lateral sclerosis, parkinsonism, dementia complex (disorder)
840452004	Classical sporadic Creutzfeldt-Jakob disease (disorder)
840464007	Dementia due to carbon monoxide poisoning (disorder)
860826006	Creutzfeldt-Jakob Disease caused by human growth hormone (disorder)
86188000	Kuru (disorder)
90099008	Subcortical leukoencephalopathy (disorder)
914921000006101	[D] Vascular dementia (disorder)
914931000006103	[D] Dementia with Lewy bodies (disorder)
914941000006108	[D] Dementia (disorder)
914951000006105	[D] Dementia in Alzheimer's disease (disorder)
9345005	Dialysis dementia (disorder)
939491000006102	[RFC] Dementia (finding)
97751000119108	Altered behavior in Alzheimer's disease (disorder)

Depression

Code (SNOMEDCT)	Term
1086471000000100	Recurrent reactive depressive episodes, severe, with psychosis (disorder)
1086661000000100	Reactive depression, prolonged single episode (disorder)
1089511000000100	Recurrent depression with current severe episode and psychotic features (disorder)
1089631000000100	Recurrent depression with current severe episode without psychotic features (disorder)
1089641000000100	Recurrent depression with current moderate episode (disorder)
1153570009	Treatment resistant depression (disorder)
1153575004	Persistent depressive disorder (disorder)
133121000119109	Severe seasonal affective disorder (disorder)
14183003	Chronic major depressive disorder, single episode (disorder)
15639000	Moderate major depression, single episode (disorder)

16264621000119100	Recurrent mild major depressive disorder co-occurrent with anxiety (disorder)
16264821000119100	Recurrent severe major depressive disorder co-occurrent with anxiety (disorder)
16264901000119100	Recurrent moderate major depressive disorder co-occurrent with anxiety (disorder)
16265061000119100	Recurrent major depressive disorder co-occurrent with anxiety in full remission (disorder)
16265301000119100	Recurrent major depressive disorder in partial remission co-occurrent with anxiety (disorder)
16266831000119100	Moderate major depressive disorder co-occurrent with anxiety single episode (disorder)
16266991000119100	Severe major depressive disorder co-occurrent with anxiety single episode (disorder)
18818009	Moderate recurrent major depression (disorder)
191459006	Senile dementia with depression (disorder)
191599006	Depression: [single major episode] or [agitated] or [endogenous (including first episode)] (disorder)
191602001	Single major depressive episode, moderate (disorder)
191604000	Single major depressive episode, severe, with psychosis (disorder)
191606003	Single major depressive episode, in full remission (disorder)
191608002	Recurrent depression: [major episode] or [endogenous] (disorder)
191610000	Recurrent major depressive episodes, mild (disorder)
191611001	Recurrent major depressive episodes, moderate (disorder)
191613003	Recurrent major depressive episodes, severe, with psychosis (disorder)
191615005	Recurrent major depressive episodes, in full remission (disorder)
191616006	Recurrent depression (disorder)
191659001	Atypical depressive disorder (disorder)
191676002	Reactive depressive psychosis (disorder)
192049004	Prolonged depressive adjustment reaction (disorder)
192080009	Chronic depression (disorder)
19527009	Single episode of major depression in full remission (disorder)
19694002	Late onset dysthymia (disorder)
231499006	Endogenous depression first episode (disorder)
231500002	Masked depression (disorder)
231504006	Mixed anxiety and depressive disorder (disorder)
247803002	Seasonal affective disorder (disorder)
2506003	Early onset dysthymia (disorder)
251000119105	Severe major depression, single episode (disorder)
2618002	Chronic recurrent major depressive disorder (disorder)
268620009	Single major depressive episode (disorder)
268621008	Recurrent major depressive episodes (disorder)

274948002	Endogenous depression - recurrent (disorder)
281000119103	Severe recurrent major depression (disorder)
288751000119101	Reactive depressive psychosis, single episode (disorder)
300706003	Endogenous depression (disorder)
30605009	Major depression in partial remission (disorder)
310496002	Moderate depression (disorder)
310497006	Severe depression (disorder)
3109008	Secondary dysthymia early onset (disorder)
319768000	Recurrent major depressive disorder with melancholic features (disorder)
320751009	Major depression, melancholic type (disorder)
321717001	Involutional depression (disorder)
33135002	Recurrent major depression in partial remission (disorder)
35489007	Depressive disorder (disorder)
357705009	Cotard's syndrome (disorder)
36170009	Secondary dysthymia late onset (disorder)
36474008	Severe recurrent major depression without psychotic features (disorder)
36923009	Major depression, single episode (disorder)
370143000	Major depressive disorder (disorder)
38451003	Primary dysthymia early onset (disorder)
38694004	Recurrent major depressive disorder with atypical features (disorder)
397701000000102	[X]Severe depressive episode without psychotic symptoms (disorder)
401211000000106	[X] Single episode major depression without psychotic symptoms (disorder)
40379007	Mild recurrent major depression (disorder)
40568001	Recurrent brief depressive disorder (disorder)
426578000	Premenstrual dysphoric disorder in remission (disorder)
42810003	Major depression in remission (disorder)
42925002	Major depressive disorder, single episode with atypical features (disorder)
455731000000100	[X] Single episode agitated depression without psychotic symptoms (disorder)
46244001	Recurrent major depression in full remission (disorder)
465441000000108	[X]Moderate depressive episode (disorder)
63412003	Major depression in full remission (disorder)
63778009	Major depressive disorder, single episode with melancholic features (disorder)
68019004	Recurrent major depression in remission (disorder)
69392006	Major depressive disorder, single episode with catatonic features (disorder)
698957003	Depressive disorder in remission (disorder)

70747007	Major depression single episode, in partial remission (disorder)
720453001	Moderately severe major depression single episode (disorder)
720454007	Minimal major depression single episode (disorder)
75084000	Severe major depression without psychotic features (disorder)
76441001	Severe major depression, single episode, without psychotic features (disorder)
764611000000100	Recurrent major depressive episodes, severe (disorder)
764631000000108	Single major depressive episode, severe (disorder)
764691000000109	Recurrent major depressive episodes, in partial remission (disorder)
764711000000106	Single major depressive episode, in remission (disorder)
78667006	Dysthymia (disorder)
832007	Moderate major depression (disorder)
83458005	Agitated depression (disorder)
87414006	Reactive depression (situational) (disorder)

Memory

Code (Read)	Term
Z7CEH13	Bad memory
Z7CEH12	Memory deficit
Z7CEH11	Memory dysfunction
Z7CEH15	Poor memory
Z7CEH14	Memory problem
E2A1000	Mild memory disturbance
Z7CE414	Memory disturbance
ZRLK.00	Graham-Kendal memory for designs test
ZRrO.11	WMS - Wechsler memory scale
ZRh6.00	Recognition memory test
Z7CEC11	Loss of memory for recent events
R00z011	[D]Memory deficit
3A90.00	Memory: count down successful
Z7CGI00	Verbal memory
3A3..00	Memory: present place
Z7CA100	Isolated memory skills
3A20.00	Memory: present time not known
3A9..00	Memory: count down
Z7CE114	No problem with memory
Z7CEN11	Invents experiences to compensate for loss of memory
3A11.00	Memory: own age known
1S21.00	Disturbance of memory for order of events
Z7CGK00	Verbal memory recall
1B1a.00	Poor auditory sequential memory

1S2..00	Memory observations
3A81.00	Memory: important person known
Z7CGA00	Procedural memory
8HTY.00	Referral to memory clinic
Z7CE.11	Observations relating to memory
7P10400	Neuropsychology test of memory
Z7CFO11	Long-term memory loss
Z7CF811	Short-term memory loss
Z7CEL00	Mild memory disturbance
Z7CFx00	Memory aided by use of labels
Z7CGH00	Autobiographical memory
Z7CFO00	Poor long-term memory
8IEn.00	Referral to memory clinic declined
ZR1n.00	Autobiographical memory interview
ZRhS100	Rivermead behavioural memory test - adult version
Z7CEA13	Impairment of primary memory
Z7CEA11	Impairment of working memory
Z7CGF00	Auditory memory
Z7CEK00	Minor memory lapses
3A6..00	Memory: present month
ZRBg.00	Everyday memory questionnaire
Z7A1500	Memory retraining
Z7CF800	Poor short-term memory
3A80.00	Memory: import.person not knwn
1B1A100	Short-term memory loss
1B1Y.00	Poor visual sequential memory
3A50.00	Memory: own DOB not known
1B1A.00	Memory loss - amnesia
Z7CGJ00	Verbal memory encoding
Z7CEJ00	Memory lapses
Z7CGC00	Visual memory
Z7CE611	Memory loss
Z7CE612	Memory gone
Z7CE615	Loss of memory
Z7CE616	LOM - Loss of memory
3A41.00	Memory: present year known
Z7CEC12	No memory for recent events
3AA1.00	Memory: address recall unsucc.
ZRrh.00	Williams memory assessment scales
ZRF..00	Fuld object memory evaluation
Z7CFz00	Memory aided by use of lists
Z7A1300	Memory skills training

8IE5000	Initial memory assessment declined
ZRkB.00	Short orientation - memory - concentration test
3A91.00	Memory: count down unsuccess.
3A7..00	Memory: important event
Z7CEB12	Poor memory for remote events
ZRqa.00	Valentine auditory memory test
3A30.00	Memory: present place not knwn
Z7CE400	Memory disturbance (& amnesia (& symptom))
3A61.00	Memory: present month known
Z7CGL00	Verbal memory for names
Z7CF100	Memory recall normal
3A4..00	Memory: present year
1B1A.13	Memory disturbance
1B1A.12	Memory loss symptom
3A5..00	Memory: own DOB
ZRrg.00	Wechsler memory scale revised
Z7CFN12	No problems with long-term memory
3A2..00	Memory: present time
3A51.00	Memory: own DOB known
Z7CGP00	Delayed verbal memory
3A40.00	Memory: present year not known
ZRhS.00	Rivermead behavioural memory test
3AA..00	Memory: address recall
3A10.00	Memory: own age not known
Z7CEF00	Temporary loss of memory
Z7CEH00	Memory impairment
ZR2X.11	Memory concentration test
3A70.00	Memory: important event not kn
ZRkC.00	Short-term memory test
Z7CE415	Loss of memory
Z7CE412	Memory loss symptom
Z7CF111	Global memory recall within normal limits
Z7CE115	Global memory recall within normal limits
Z7CE113	Memory intact
Z7CF700	Short-term memory within normal limits
ZRrO.00	Wechsler memory scale
E2A1100	Organic memory impairment
3A31.00	Memory: present place known
3A60.00	Memory: present month not knwn
3A8..00	Memory: important person
Z7CEM00	Distortion of memory
Z7CFw00	Memory aided by use of diary

38C1500	Initial memory assessment
ZRh6.11	RMT - Recognition memory test
3A21.00	Memory: present time known
3A...11	Memory assessment
ZR2X.13	Information-memory-concentration test
ZRhS.11	RBMT - Rivermead behavioural memory test
9Nk1.00	Seen in memory clinic
Z7CF.00	Observations of memory performance
ZR1n.12	AMI - Autobiographical memory interview
ZD11300	Auditory memory therapy

Fall

Read code	Read Term
U10H.00	[X]Other fall from one level to another
ZV71B00	[V]Examination and observation following a fall
U10H600	[X]Other fall frm one level to anoth occ indust/constr area
T170600	MVTA-fall down bus stairs - pedal cyclist injured
U10Hy00	[X]Other fall frm one levl to anothr occ at oth specif plce
TCyz.00	Other accidental fall NOS
TN70.00	Injury ?accidental, fall from residential premises
TN72.00	Injury ?accidental, fall from natural site
TC4yz00	Other fall from one level to another NOS
TC4..00	Other fall from one level to another
U10z600	[X]Unspecified fall occurrn at industrial/construction area
TC4y.00	Other fall from one level to another
U10zy00	[X]Unspecified fall, occurrence at other specified place
T170100	MVTA-fall down bus stairs - motor vehicle passenger injured
T170y00	MVTA-fall down bus stairs - other specified person injured
TN7z.00	Injury ?accidental, fall from high place NOS
TN7..00	Injury ?accidental, fall from high place
U10zz00	[X]Unspecified fall, occurrence at unspecified place
U10Hz00	[X]Othr fall frm one level to anothr occurrn at unspec plce
U10H500	[X]Other fall frm one level to anothr occ at trde/serv area
TCy..00	Other falls
TC...00	Accidental falls
615B.11	IUD fallen out
TCz..00	Accidental falls NOS
16D1.00	Recurrent falls
T183600	MVTA-fall from moving MV - pedal cyclist injured
T253.00	MVNTA - fall from moving motor vehicle, except off-road MV
U10z.00	[X]Unspecified fall
U10J600	[X]Other fall on same level, occurring at induct/construct area

U10H300	[X]Other fall from one level to another occ sport/athlet area
U10H200	[X]Other fall from one level to anothr, sch inst/pub adm area
U10z000	[X]Unspecified fall, occurrence at home
U10H400	[X]Othr fall from one level to anothr occurrn street/h'way
U10H000	[X]Other fall from one level to another, occurrence at home
T171.00	MVTA - fall from car in street while boarding/alighting
T171700	MVTA-fall from car in street - pedestrian injured
R200.12	[D] Geriatric fall
U131.00	[X]Drowning and submersion following fall into bath-tub
T189100	MVTA-obj falling on mov MV - motor vehicle passenger injured
U10J000	[X]Other fall on same level, occurrence at home
U10J100	[X]Other fall on same level, occurrnce in resident instit'n
U10z100	[X]Unspecified fall, occurrence in residential institution
U10z400	[X]Unspecified fall, occurrence on street and highway
U10J.00	[X]Other fall on same level
T183.00	MVTA - fall from motor vehicle while in motion
T170400	MVTA-fall down bus stairs - occupant of tram injured
T170.00	MVTA - fall downstairs of motor bus while board/alighting

Sleep

Read code	Read Term
Fy03.00	Sleep apnoea
Fy03.11	Obstructive sleep apnoea
H5B..00	Sleep apnoea
H5B0.00	Obstructive sleep apnoea
R005100	[D]Insomnia with sleep apnoea
R005300	[D]Hypersomnia with sleep apnoea
R005311	[D]Sleep apnoea syndrome
R005312	[D]Syndrome sleep apnoea
C380200	Extreme obesity with alveolar hypoventilation
C38y.11	Pickwickian syndrome
C38y000	Pickwickian syndrome
Z1M..00	Sleep and rest interventions
R005800	[D]Sleep dysfunction with sleep stage disturbance
R005100	[D]Insomnia with sleep apnoea
Eu51z11	[X]Emotional sleep disorder NOS
38D0.00	Pittsburgh sleep quality index
E274D11	Restless sleep
8HTn.00	Referral to sleep clinic
8Q0..00	Sleep management
Fy0..00	Sleep disorders

R005311	[D]Sleep apnoea syndrome
9Nk0.00	Seen in sleep clinic
E274000	Unspecified non-organic sleep disorder
R005000	[D]Sleep disturbance, unspecified
E274C00	Other sleep stage or arousal dysfunction
ZV75312	[V]Screening for sleeping sickness
Fy00.00	Disorders of initiating and maintaining sleep
Fy02.00	Disorders of the sleep-wake schedule
R005.00	[D]Sleep disturbances
1BX2.00	Sleeping pattern
R005z00	[D]Sleep dysfunction NOS
Fy03.00	Sleep apnoea
E274F00	Inversion of sleep rhythm
R005500	[D]Sleep rhythm inversion
R005.12	[D]Sleep rhythm problems
1B1Q.00	Poor sleep pattern
1BX9.00	Light sleep
Fyu5800	[X]Other sleep disorders
1BX..00	Sleep observations
1BX1.00	Excessive sleep
7065800	Sleep studies
Eu51213	[X]Psychogenic inversion of sleep rhythm
Z1M1.00	Disturbing sleep
7065A00	Sleep studies NEC
R005900	[D]Sleep dysfunction with arousal disturbance
9b9Y.00	Sleep studies - specialty
8G9B.00	Sleep hygiene behaviour education
Eu51000	[X]Nonorganic insomnia
1B1B100	Middle insomnia
1B1B200	Late insomnia
E274100	Transient insomnia
R005200	[D]Insomnia NOS
E274200	Persistent insomnia
R005.11	[D]Insomnia - symptom
1B1B.11	C/O - insomnia
1B1B000	Initial insomnia
E274111	Insomnia NOS
E274.12	Insomnia due to nonorganic sleep disorder
1B1B.00	Cannot sleep - insomnia
38D1.00	Insomnia severity index

Anxiety

Code (ICD)	Term
F41.0	Panic disorder [episodic paroxysmal anxiety]
F41.1	Generalized anxiety disorder
F41.2	Mixed anxiety and depressive disorder
F41.3	Other mixed anxiety disorders
F41.8	Other specified anxiety disorders
F41.9	Anxiety disorder, unspecified

Code (SNO code)	Term
48694002	Anxiety
21897009	Generalized anxiety disorder
371631005	Panic disorder
231504006	Mixed anxiety and depressive disorder
197480006	Anxiety disorder

FOG

Code (SNOMEDCT)	Term
443544006	Freezing of gait
387603000	Impaired balance
129839007	At risk of falls
22325002	Gait abnormality

Hearing Loss

Code (ICD 10)	Term
H90.0	Conductive hearing loss, bilateral
H90.1	Conductive hearing loss, unilateral with unrestricted hearing on the contralateral side
H90.2	Conductive hearing loss, unspecified
H90.3	Sensorineural hearing loss, bilateral
H90.4	Sensorineural hearing loss, unilateral with unrestricted hearing on the contralateral side
H90.5	Sensorineural hearing loss, unspecified
H90.6	Mixed conductive and sensorineural hearing loss, bilateral
H90.7	Mixed conductive and sensorineural hearing loss, unilateral with unrestricted hearing on the contralateral side
H90.8	Mixed conductive and sensorineural hearing loss, unspecified
H91.0	Ototoxic hearing loss
H91.1	Presbycusis
H91.2	Sudden idiopathic hearing loss
H91.3	Deaf mutism, not elsewhere classified
H91.8	Other specified hearing loss

H91.9	Hearing loss, unspecified

Supplementary Method 8: Alzheimer's disease (AD) and Parkinson's disease (PD) specific symptom code definition

MCI

Code (SNOMED CT)	Term
386806002	Cognitive decline
386805003	Mild cognitive disorder
386805003	Mild cognitive impairment
386806002	Cognitive impairment
407631005	GDS level 3 - mild cognitive decline
407630006	GDS level 2 - very mild cognitive decline
1.05E+15	Mild cognitive impairment review
1.95E+15	Dementia stage at diagnosis - early (mild)
1.93E+15	Referral to dementia early intervention service
48167000	[X]Other amnesia
48167000	Memory loss - amnesia
48167000	Amnesia symptom
48167000	Amnesia
48167000	Memory loss
48167000	Loss of memory
42176003	Amnesia for recent events
42176003	Loss of memory for recent events
836301008	Amnestic mild cognitive disorder
836301008	aMCI - amnestic mild cognitive impairment
443265004	Cognitive disorder
72440003	Disorientated in place
19657006	Disorientated in time
55533009	Forgetful
247595006	Forgets recent activities
247596007	Forgets what has just done
247600002	Forgets what has just heard
247597003	Forgets what has just said
247664009	Getting lost
407630006	GDS level 2 - very mild cognitive decline
407631005	GDS level 3 - mild cognitive decline
283902008	Has delayed recall
386806002	Cognitive impairment
386806002	Cognitive decline
386806002	Impaired cognition

386806002	Impaired cognition
386806002	Cognitive disturbance
386806002	Cognitive dysfunction
386806002	Cognitive deficit
386807006	Memory deficit
386807006	Memory impairment
386807006	Memory disturbance
386807006	Memory dysfunction
386807006	Impaired memory
386807006	Memory problem
386807006	Poor memory
386807006	Bad memory
386807006	Disturbance of memory
225038006	Memory lapses
408902006	Memory loss care assessment
165308007	Memory: address recall unsuccessful
165298000	Memory: important event not known
165298000	Memory: important event not known
165295002	Memory: present month not known
165295002	Memory: present month not known
165286005	Memory: present place not known
165283002	Memory: present time not known
165289003	Memory: present year not known
386805003	Mild cognitive disorder
386805003	Mild cognitive impairment
192071009	Mild memory disturbance
110352000	Minimal cognitive impairment
225037001	Minor memory lapses
247592009	Short-term memory loss
247592009	Poor short-term memory
247592009	Short term memory loss
8240000000000	Referral to memory assessment service
415276009	Referral to memory clinic
51921000	Retrograde amnesia
112077003	Spatial disorientation
285208000	Unable to recall random address at five minutes

Tremor

Code (ICD 10)	Term
G25.0	Essential tremor
G25.1	Drug-induced tremor
G25.2	Other specified forms of tremor

R25.1	Tremor, unspecified

Code (SNOMEDCT)	Term
609558009	Essential tremor
427451007	Dystonic tremor
26079004	Tremor (finding)
25082004	Resting tremor (finding).
30721006	Action tremor (finding).
308909003	Parkinson Tremor

Supplementary Method 9: Polygenic risk scores (PRS) calculation

We used the pre-calculated UKB standard PRS (category 301) which contains 39 PRS. We first compared AD (field ID 20206) and PD (field ID 20260) with control cases, which are non-disease Caucasian and genetic sex matched with self-reported sex. Then we compare the 5 clusters other 37 PRS score to generate the PRS heatmap.

The UKB standard PRS were calculated using a systematic approach, integrating data from external genome-wide association studies (GWAS) to assess genetic predispositions for various traits and diseases. The key steps in this process include:
1. Selection of External GWAS Data: For each trait or disease, relevant external GWAS datasets were identified. These studies provided summary statistics, including effect sizes and p-values for single nucleotide polymorphisms (SNPs) associated with the traits of interest.
2. Meta-Analysis of GWAS Data: The selected GWAS datasets were combined through meta-analysis to derive more robust effect size estimates for each SNP, enhancing statistical power by integrating findings from multiple studies.
3. Quality Control and SNP Selection: SNPs underwent stringent quality control measures to ensure data integrity. Criteria such as minor allele frequency thresholds, imputation quality scores, and consistency across datasets were applied. Additionally, linkage disequilibrium (LD) pruning or clumping methods were employed to select independent SNPs, minimizing redundancy due to correlated genetic variants.
4. Calculation of Polygenic Risk Scores
For details, please refer to the original paper [1].

Supplementary Method 10: single-nucleotide polymorphism (SNP) extraction, quality control (QC) and visualisation

We select patient who are Caucasian people in UK Biobank and the self-report sex are matched with genetic sex. We detail our process for analysing specific SNPs associated with AD and PD:

Genotype Data QC Processing:

Stringent quality control measures were applied, including:

- Minor allele frequency (MAF) is greater than 0.01
- Minor allele count is greater than 100
- Missing call rate for variant is not exceeding 0.1
- Missing call rate for sample is not exceeding 0.1
- Hardy-Weinberg equilibrium exact test p-value for the variant is greater than 1e-15

SNP Selection:

For AD, we focused on SNPs within the genes *APOE4* rs429358 [2], *APOE2* rs7412 [2], *TREM2* rs143332484 [3], and *ABCA7* rs3764650 [4], which have been implicated in AD susceptibility. For PD, we examined SNPs in the genes *APOE4* rs429358 [5], *APOE2* rs7412 [5], *LRRK2* rs34637584, *PRKN* rs34424986, and *GBA1* rs2230288 [6], known for their associations with PD risk.

Carrier frequency definition, Analysis and Visualisation:

Genotypes were coded using a dominant (carrier) model as follows: individuals homozygous for the reference allele were coded as 0 (non-carriers), while individuals heterozygous or homozygous for the alternate allele were coded as 1 (carriers). This binary coding distinguished carrier from non-carrier status for each variant.

For each cluster, carrier prevalence was calculated as the proportion of individuals carrying at least one alternate allele (coded as 1) among all individuals in that cluster.

To assess relative enrichment across clusters, we computed a carrier enrichment ratio by dividing the cluster-specific carrier prevalence by the corresponding general population General population minor allele frequencies (MAF). These ratios were visualised using heatmaps to facilitate comparison of variant-specific enrichment patterns across clusters. General population MAF for the selected variants were obtained from the NCBI ALFA database using European ancestry data.

Reference

1. Thompson, D.J., Wells, D., Selzam, S., Peneva, I., Moore, R., Sharp, K., Tarran, W.A., Beard, E.J., Riveros-Mckay, F., Giner-Delgado, C. and Palmer, D., 2022. UK Biobank release and systematic evaluation of optimised polygenic risk scores for 53 diseases and quantitative traits. *MedRxiv*, pp.2022-06.
2. Kulminski AM, Shu L, Loika Y, He L, Nazarian A, Arbeev K, Ukraintseva S, Yashin A, Culminskaya I. Genetic and regulatory architecture of Alzheimer's disease in the APOE region. Alzheimer's & Dementia: Diagnosis, Assessment & Disease Monitoring. 2020;12(1):e12008.
3. Zhou SL, Tan CC, Hou XH, Cao XP, Tan L, Yu JT. TREM2 variants and neurodegenerative diseases: a systematic review and meta-analysis. Journal of Alzheimer's Disease. 2019 Apr 8;68(3):1171-84.
4. Liu G, Li F, Zhang S, Jiang Y, Ma G, Shang H, Liu J, Feng R, Zhang L, Liao M, Zhao B. Analyzing large-scale samples confirms the association between the ABCA7 rs3764650 polymorphism and Alzheimer's disease susceptibility. Molecular neurobiology. 2014 Dec;50:757-64.
5. Kim R, Park S, Yoo D, Jun JS, Jeon B. Association of physical activity and APOE genotype with longitudinal cognitive change in early Parkinson disease. Neurology. 2021 May 11;96(19):e2429-37.
6. Pitz V, Makarious MB, Bandres-Ciga S, Iwaki H, Singleton AB, Nalls M, Heilbron K, Blauwendraat C. Analysis of rare Parkinson's disease variants in millions of people. NPJ Parkinson's disease. 2024 Jan 8;10(1):11.
7. L. Phan YJ, H. Zhang, W. Qiang, E. Shekhtman, D. Shao, D. Revoe, R. Villamarin, E. Ivanchenko, M. Kimura, Z. Y. Wang, L. Hao, N. Sharopova, M. Bihan, A. Sturcke, M. Lee, N. Popova, W. Wu, C. Bastiani, M. Ward, J. B. Holmes, V. Lyoshin, K. Kaur, E. Moyer, M. Feolo, and B. L. Kattman. ALFA: Allele Frequency Aggregator. *National Center for Biotechnology Information, US National Library of Medicine* 2020.

Supplementary Figures

Supplementary Figure 1

Flow diagram of Alzheimer's disease (AD) patient selection in the Clinical Practice Research Datalink (CPRD) cohort. GP = general practice; HES = Hospital Episode Statistics.

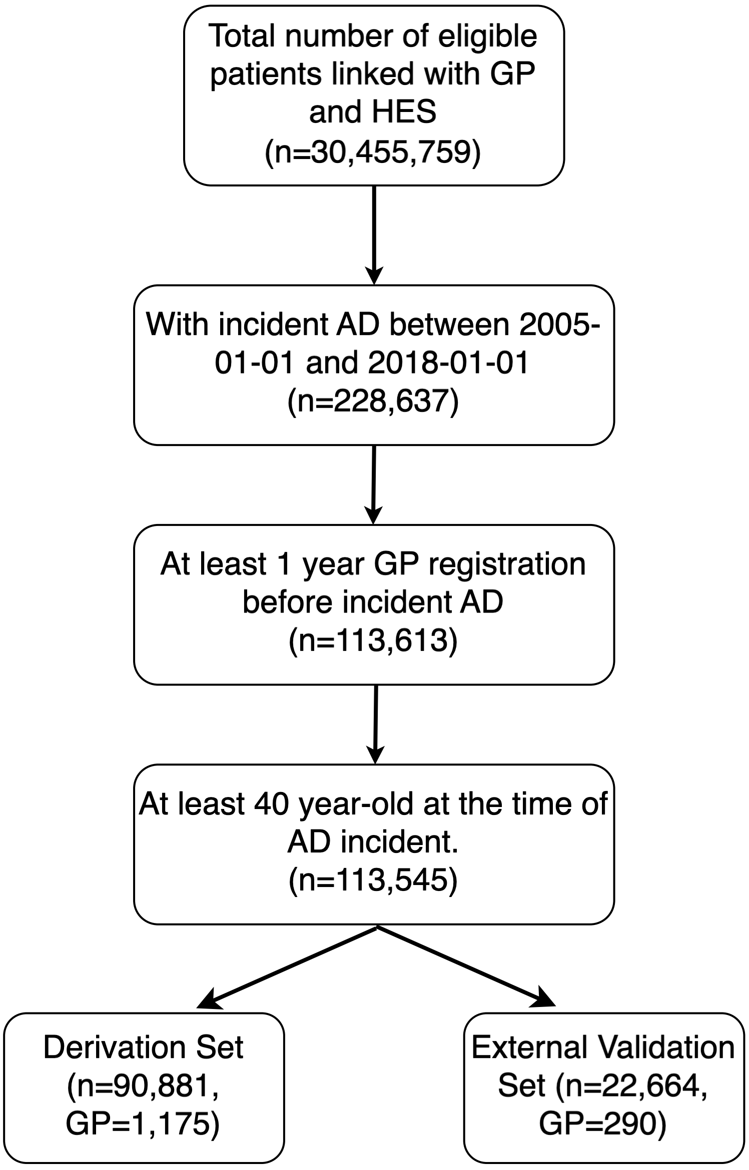

Supplementary Figure 2

Flow diagram of Alzheimer's disease (AD) patient selection in the UK Biobank (UKB) cohort. GP = general practice

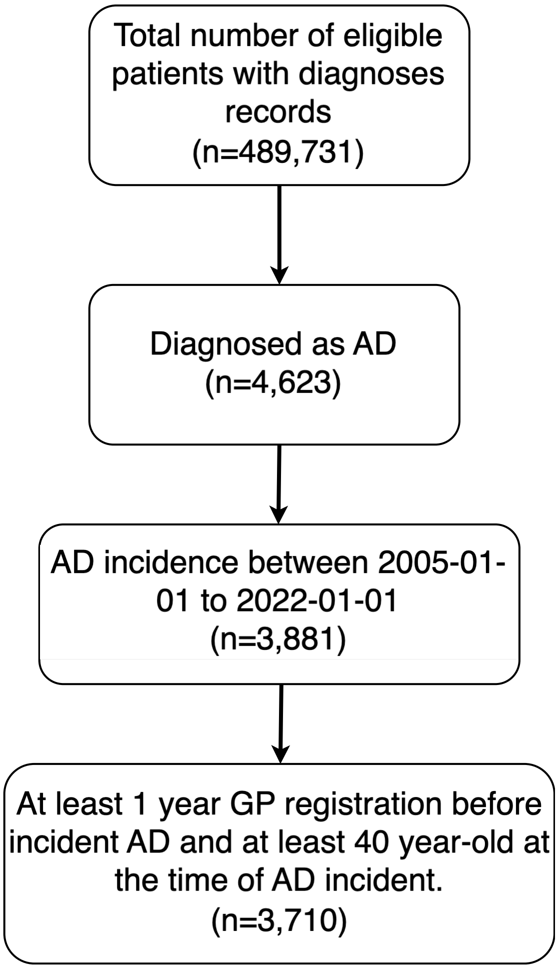

Supplementary Figure 3

Flow diagram of Parkinson's disease (PD) patient selection in the Clinical Practice Research Datalink (CPRD) cohort. GP = general practice; HES = Hospital Episode Statistics.

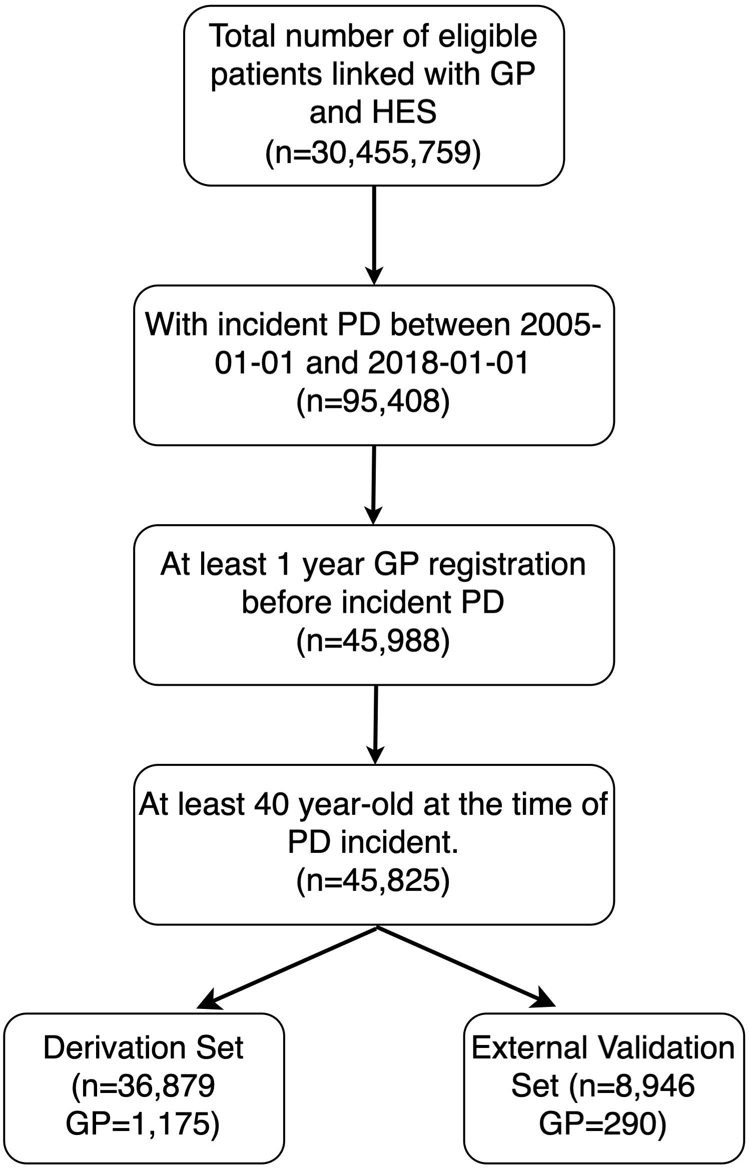

Supplementary Figure 4

Flow diagram of Parkinson's disease (PD) patient selection in the UK Biobank (UKB) cohort. GP = general practice.

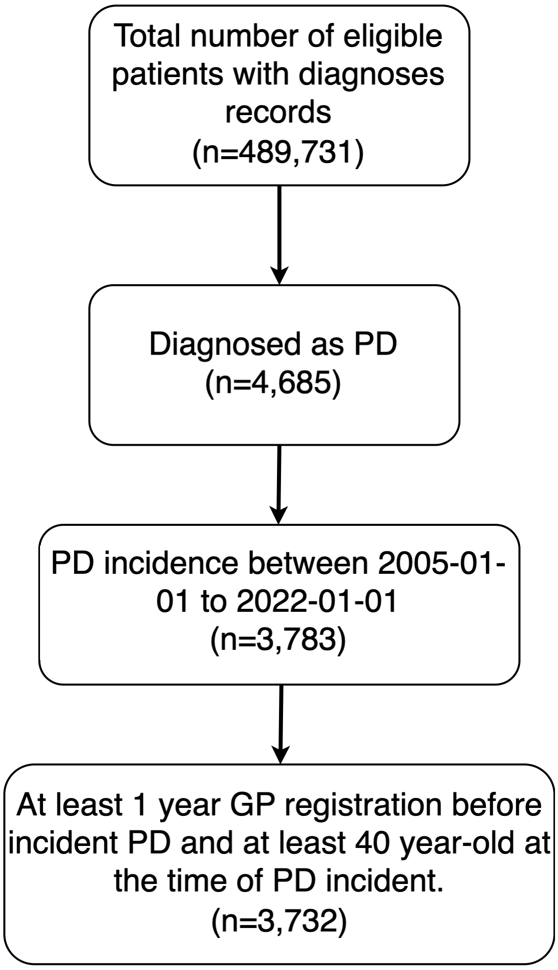

Supplementary Figure 5

t-SNE visualisation of Alzheimer's disease (AD) patient embeddings for (a) Clinical Practice Research Datalink (CPRD) derivation, (b) CPRD validation, and (c) UK Biobank datasets (K = 5 clusters). Each dot represents one patient, colour-coded by cluster membership.

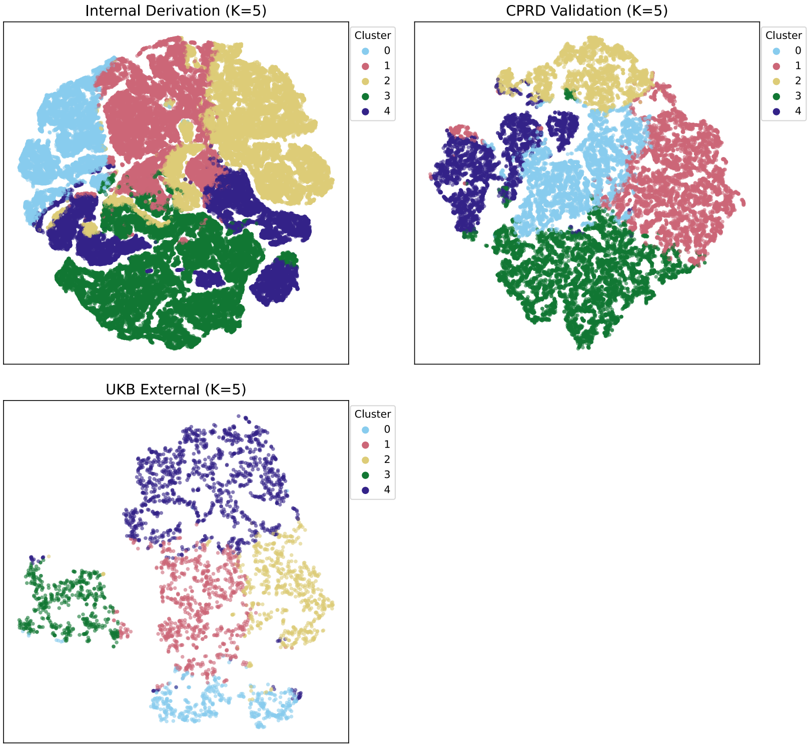

Supplementary Figure 6

t-SNE visualisation of Parkinson's disease (PD) patient embeddings for (a) Clinical Practice Research Datalink (CPRD) derivation, (b) CPRD validation, and (c) UK Biobank datasets (K = 5 clusters). Each dot represents one patient, colour-coded by cluster membership.

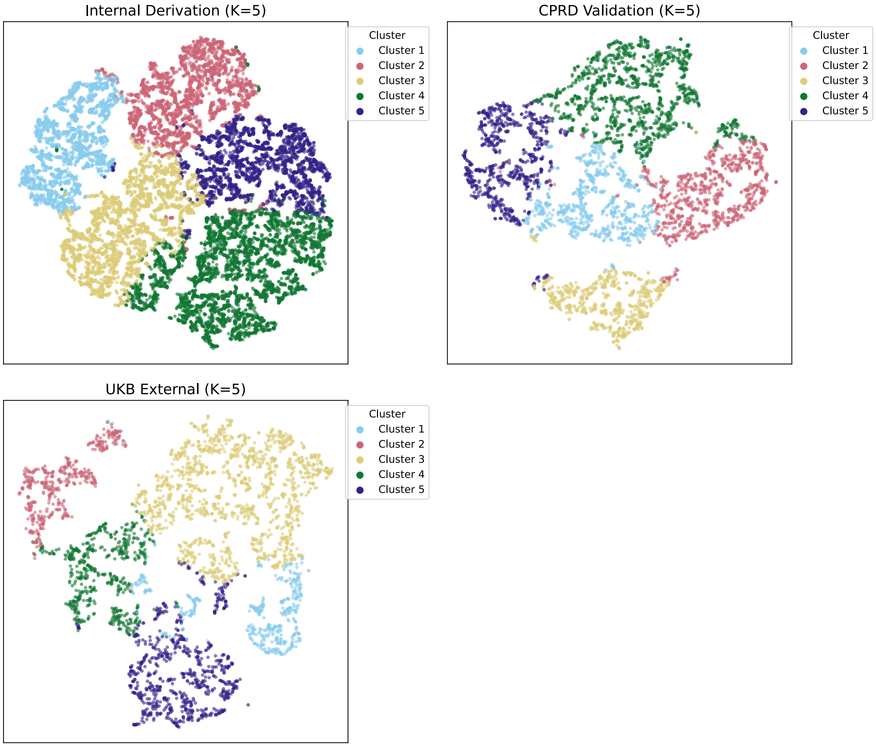

Supplementary Figure 7

Alzheimer’s Disease (AD) Clinical Practice Research Datalink (CPRD) deviation dataset cluster and age distribution (N= 90,881)

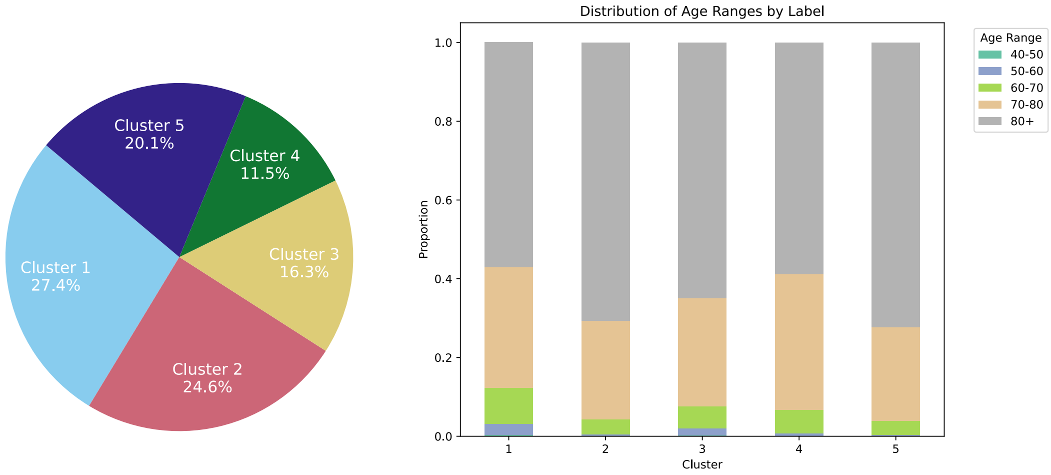

Supplementary Figure 8

Alzheimer’s Disease (AD) UK Biobank dataset cluster and age distribution (N=3,710).

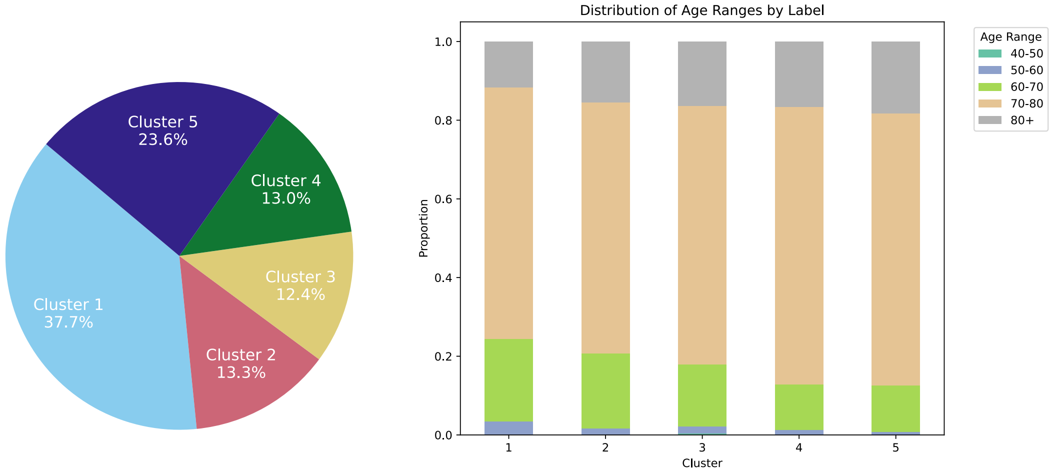

Supplementary Figure 9

Parkinson's Disease (PD) Clinical Practice Research Datalink (CPRD) deviation dataset cluster and age distribution (N= 36,879).

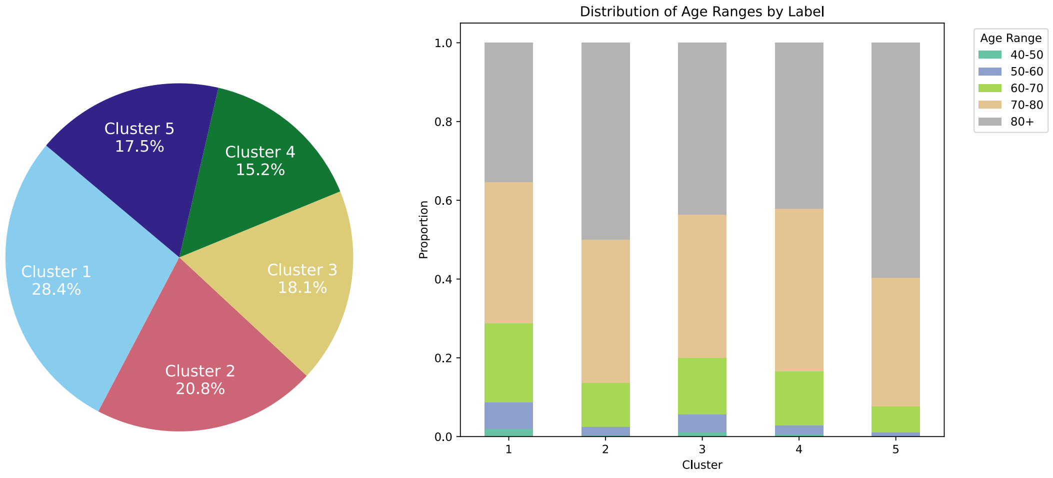

Supplementary Figure 10

Parkinson's Disease (PD) UK Biobank dataset cluster and age distribution (N= 3,732).

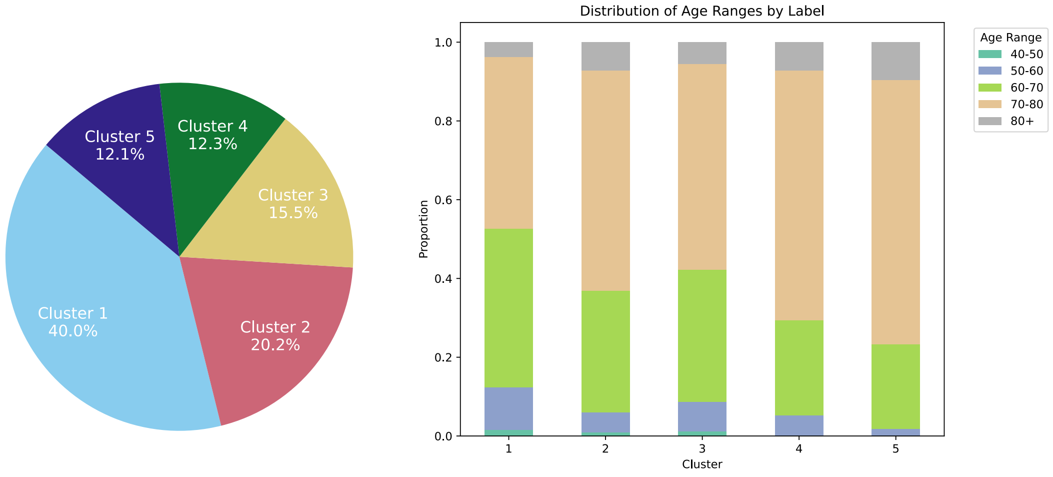

Supplementary Figure 11

Alzheimer's Disease (AD) UK Biobank 5-year mortality and hospitalisation. Solid lines represent the estimated survival or hospitalisation rates, and shaded regions represent the 95% confidence intervals (95% CI).

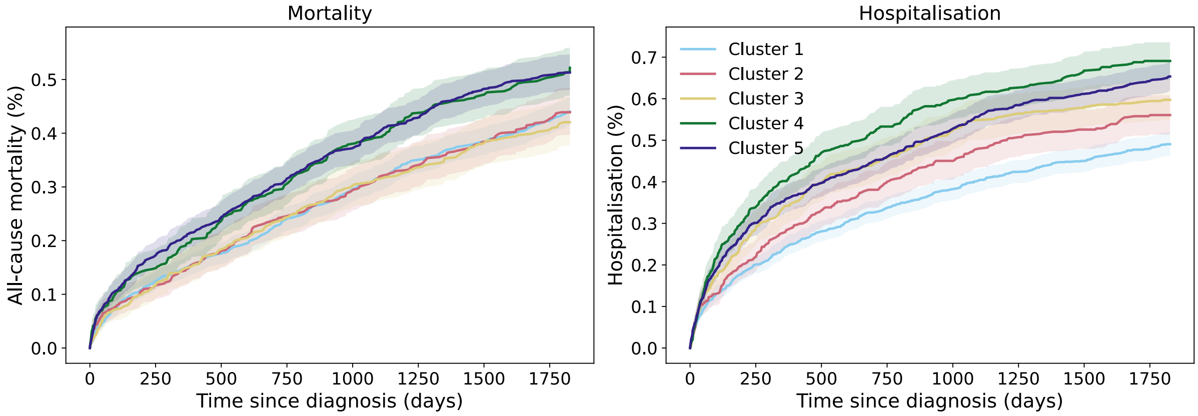

Supplementary Figure 12

Parkinson's Disease (PD) UK Biobank 5-year mortality and hospitalisation. Solid lines represent the estimated survival or hospitalisation rates, and shaded regions represent the 95% confidence intervals (95% CI).

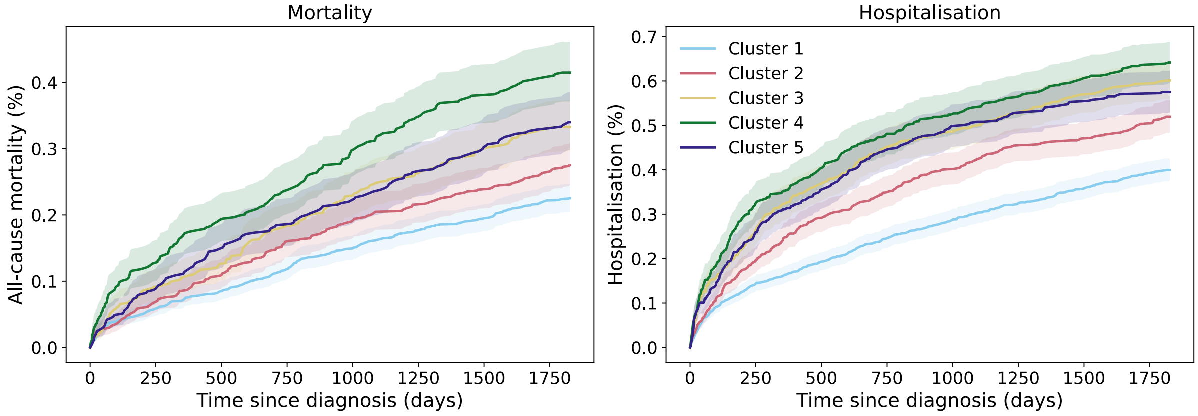

Supplementary Figure 13

Alzheimer's Disease (AD) subtype–specific discriminative comorbidity profiles.
Radar plots summarise the top five diagnosis codes and top five medication codes that most strongly distinguish each AD subtype. Discriminative strength was quantified using the weighted discriminative score (WDS).

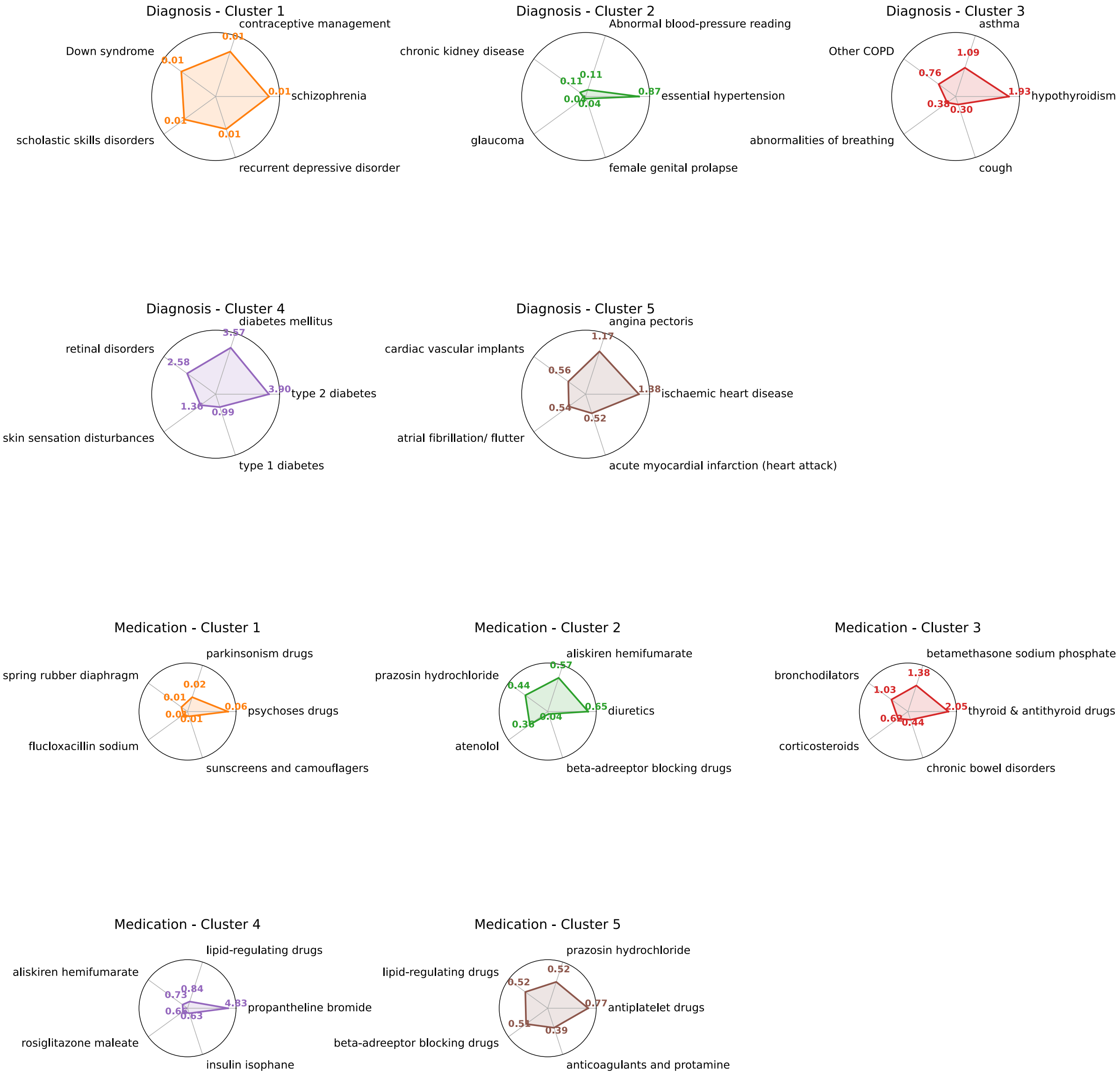

Supplementary Figure 14

Parkinson’s disease (PD) subtype–specific discriminative comorbidity profiles.
Radar plots summarise the top five diagnosis codes and top five medication codes that most strongly distinguish each PD subtype. Discriminative strength was quantified using the weighted discriminative score (WDS)

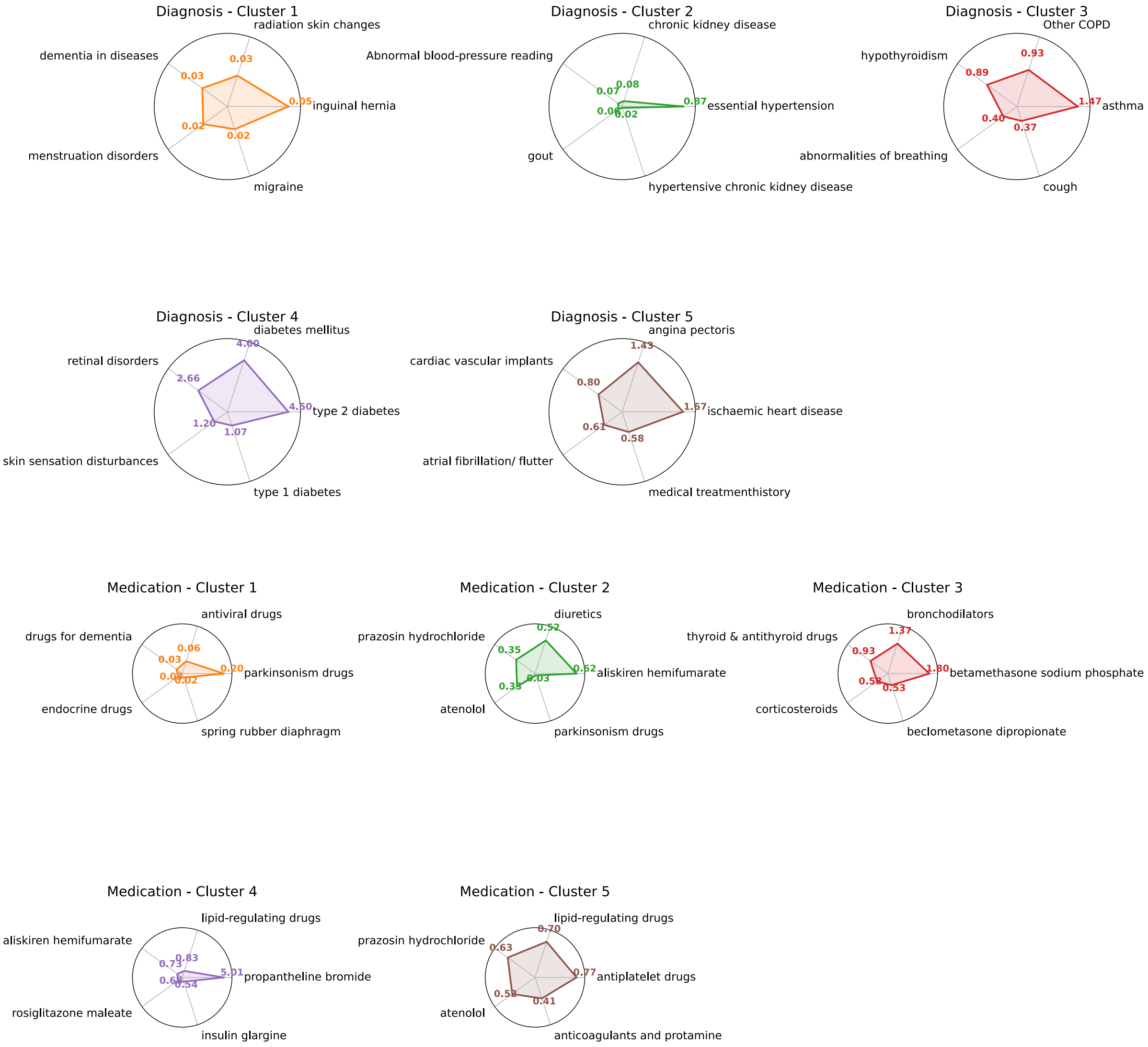

Supplementary Figure 15

Alzheimer's Disease (AD) UK Biobank disease comorbidity, showing diseases with more than 15% variance across clusters. Numbers in the heatmap represent the percentage of individuals within each cluster who have the corresponding diagnosis, normalised by the total number of individuals in that cluster.

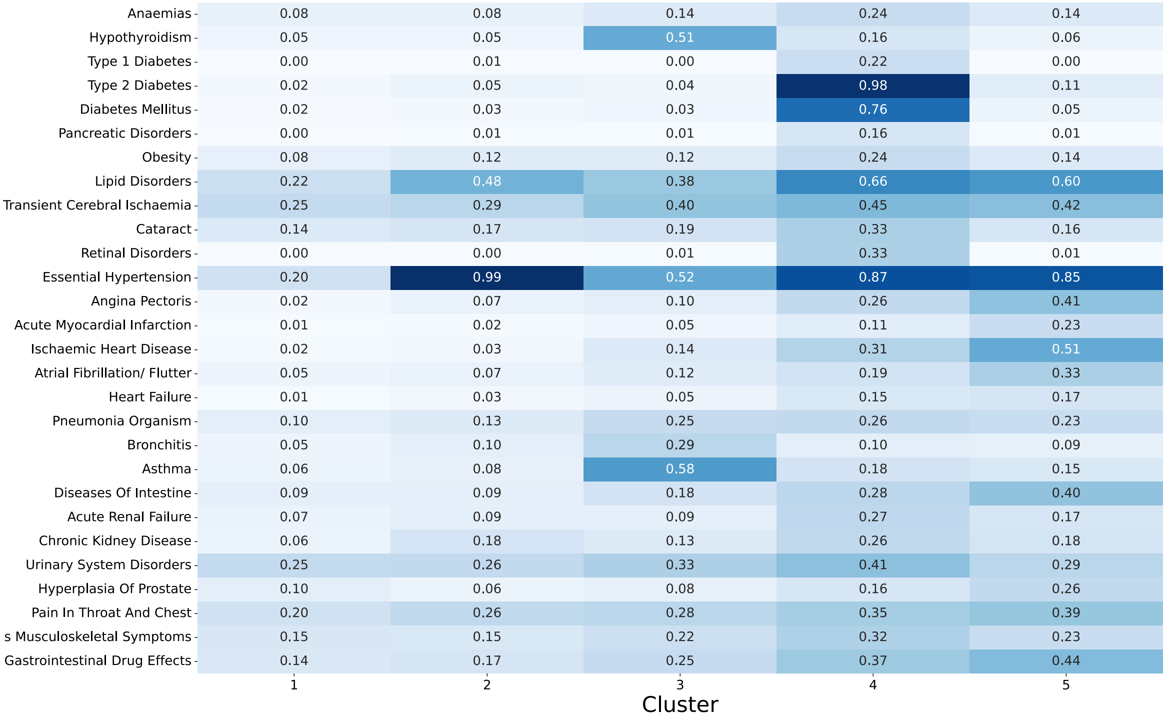

Supplementary Figure 16

Parkinson's Disease (PD) UK Biobank disease comorbidity, showing diseases with more than 15% variance across clusters. Numbers in the heatmap represent the percentage of individuals within each cluster who have the corresponding diagnosis, normalised by the total number of individuals in that cluster.

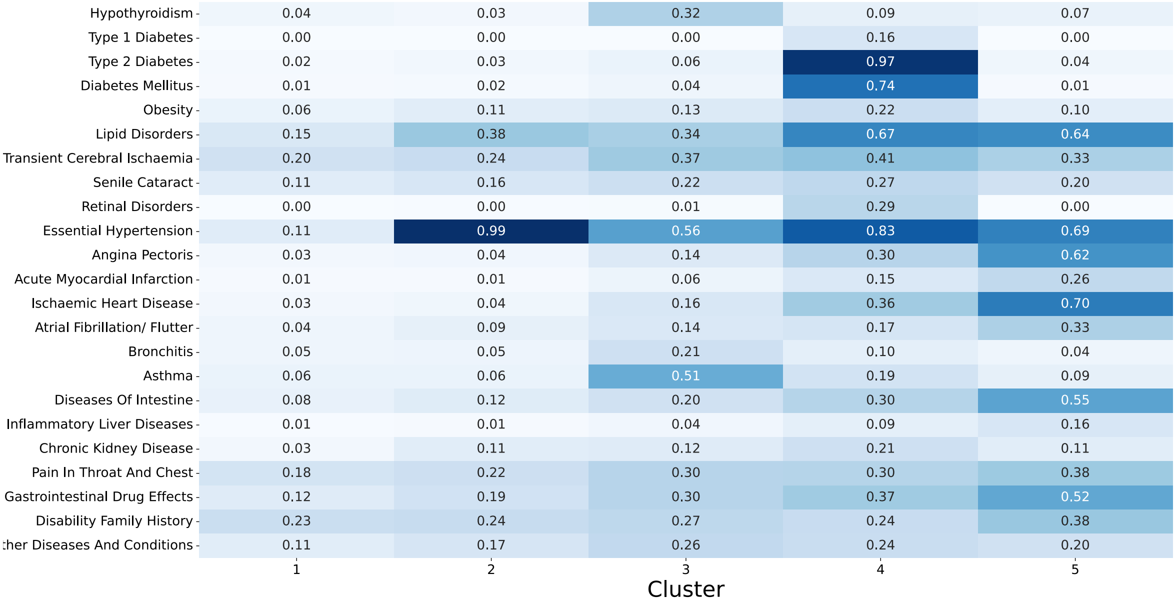

Supplementary Figure 17

Ten-year prevalence (five years pre- and five years post-diagnosis) of Alzheimer's Disease (AD) Depression prevalence.

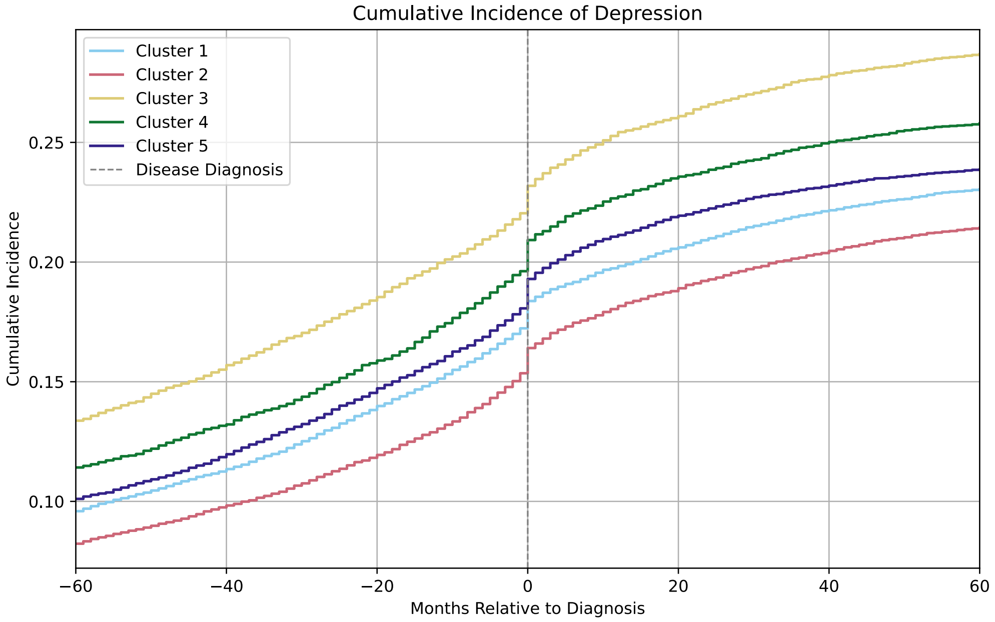

Supplementary Figure 18

Ten-year prevalence (five years pre- and five years post-diagnosis) of Alzheimer's Disease (AD) fall prevalence.

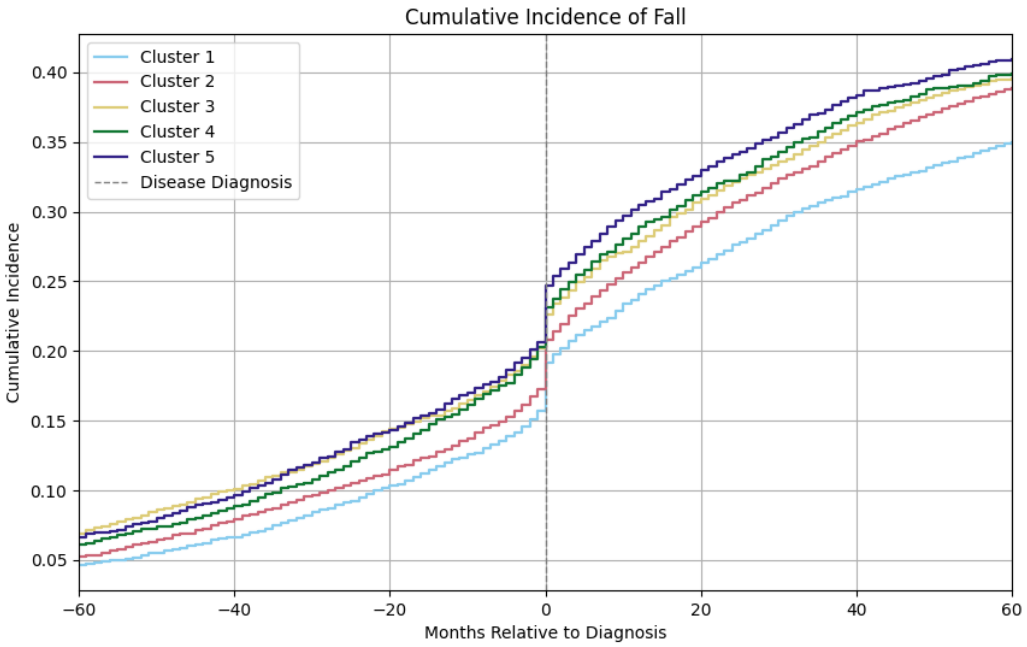

Supplementary Figure 19

Ten-year prevalence (five years pre- and five years post-diagnosis) of Parkinson's Disease (PD) depression prevalence

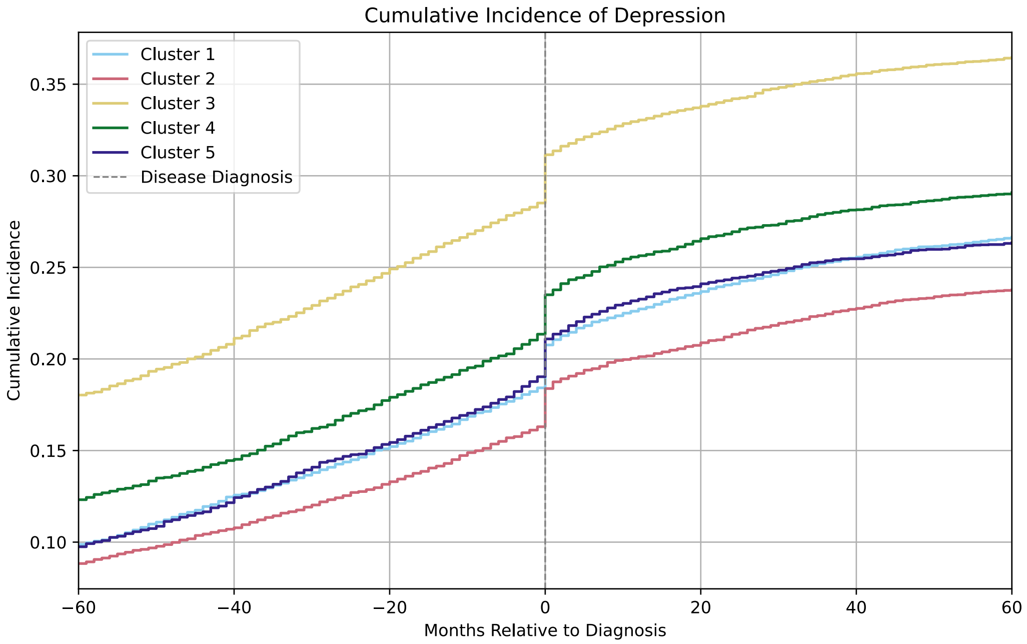

Supplementary Figure 20

Ten-year prevalence (five years pre- and five years post-diagnosis) of Parkinson's Disease (PD) tremor prevalence

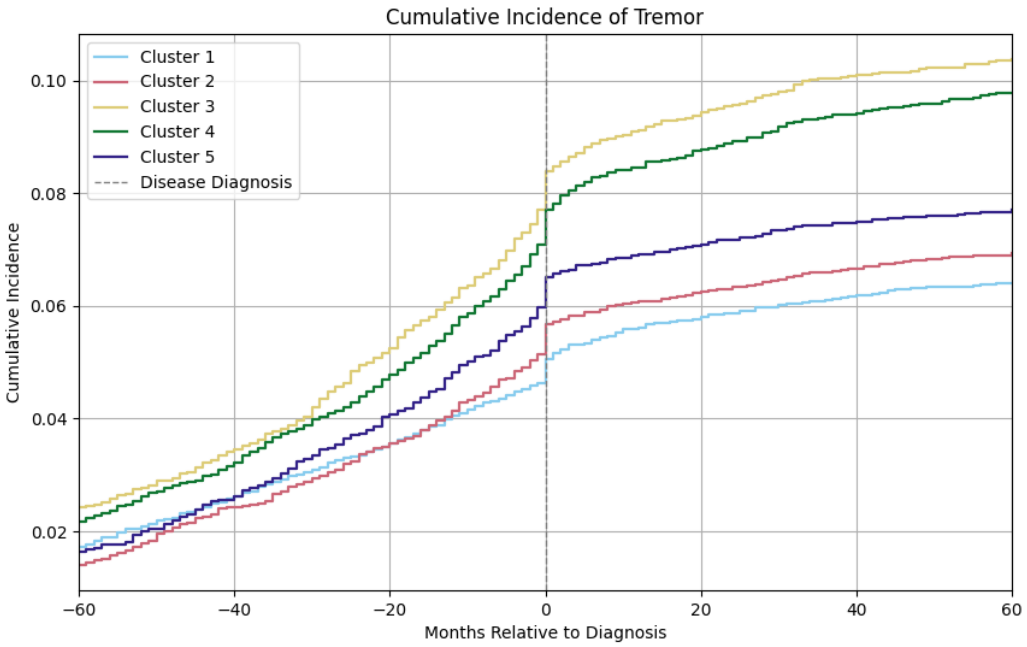

Supplementary Figure 21

Ten-year prevalence (five years pre- and five years post-diagnosis) of Parkinson's Disease (PD) dementia prevalence

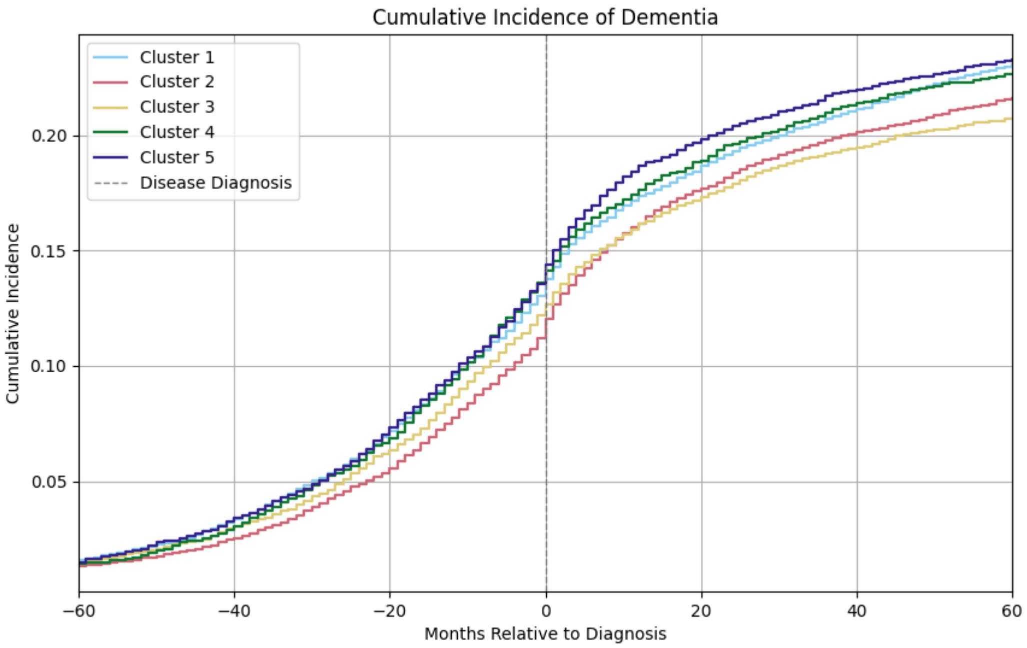

Supplementary Figure 22

Heatmap of additional Alzheimer's disease (AD) polygenic risk scores (PRS) across the five identified clusters in the UK Biobank dataset. This heatmap displays the mean values of 13 additional AD PRS across the five AD clusters identified in the UK Biobank dataset. For each PRS, the colour scale represents the average standardized score within each cluster, illustrating distinct genetic enrichment patterns across subtypes. Values shown represent mean PRS per cluster.

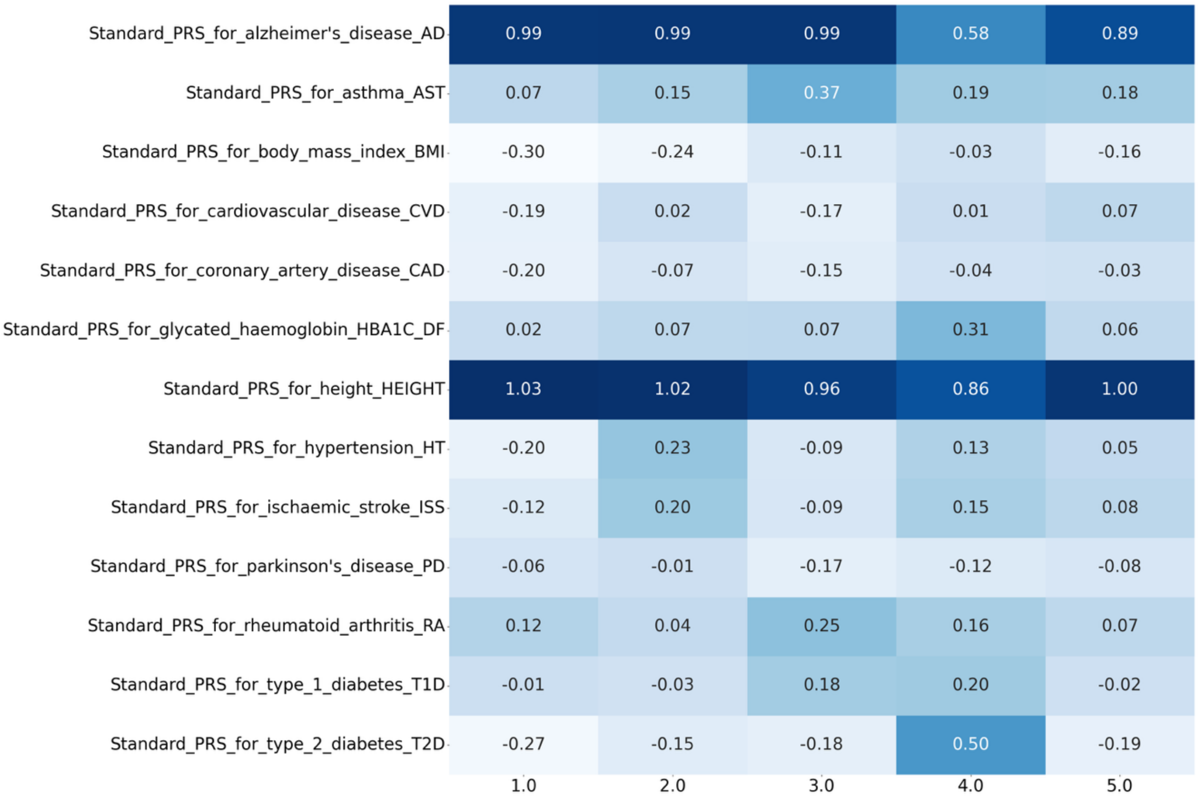

Supplementary Figure 23

Heatmap of additional Parkinson’s disease (PD) polygenic risk scores (PRS) across the five identified clusters in the UK Biobank dataset. This heatmap displays the mean values of 13 additional PD PRS across the five PD clusters identified in the UK Biobank dataset. For each PRS, the colour scale represents the average standardized score within each cluster, illustrating distinct genetic enrichment patterns across subtypes. Values shown represent mean PRS per cluster.

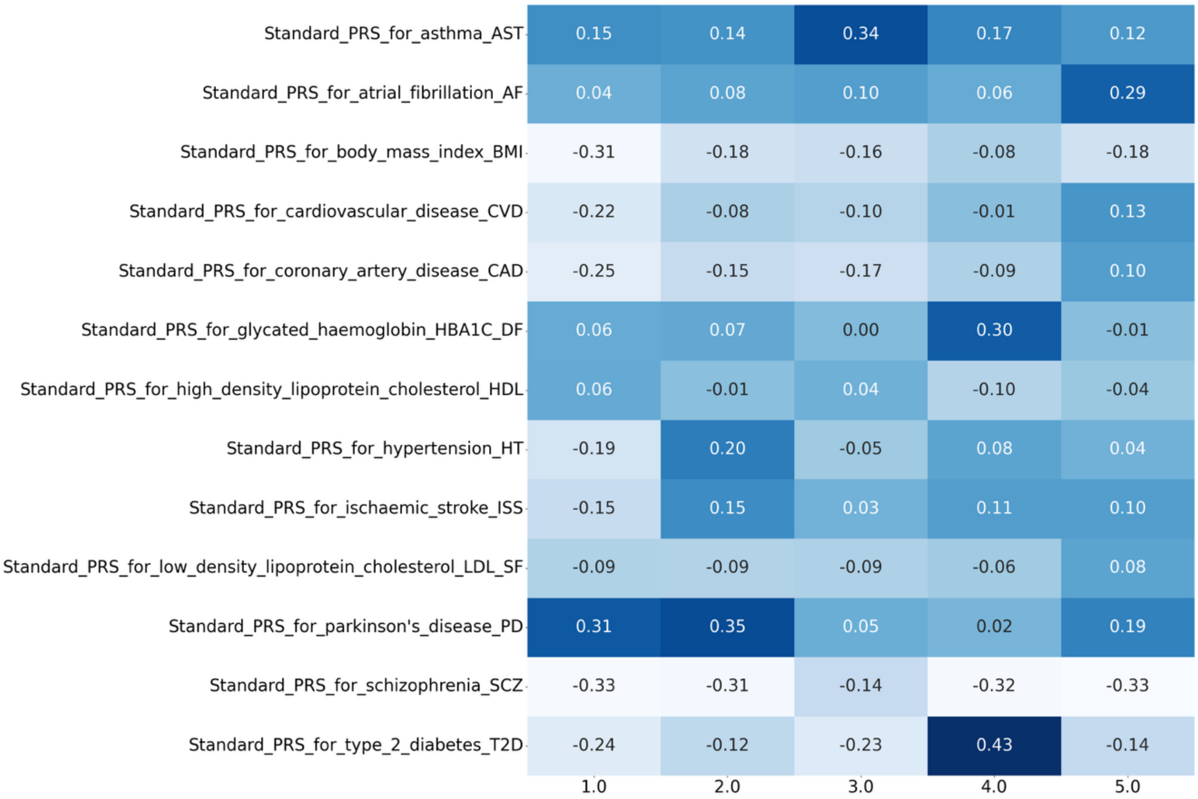

Supplementary Figure 24

Alzheimer's Disease (AD) carrier enrichment heatmap. This heatmap shows the carrier enrichment ratio for AD-associated single-nucleotide polymorphisms (SNPs) across the five AD clusters. The ratio was calculated as the cluster-specific carrier prevalence divided by the corresponding general population minor allele frequency.

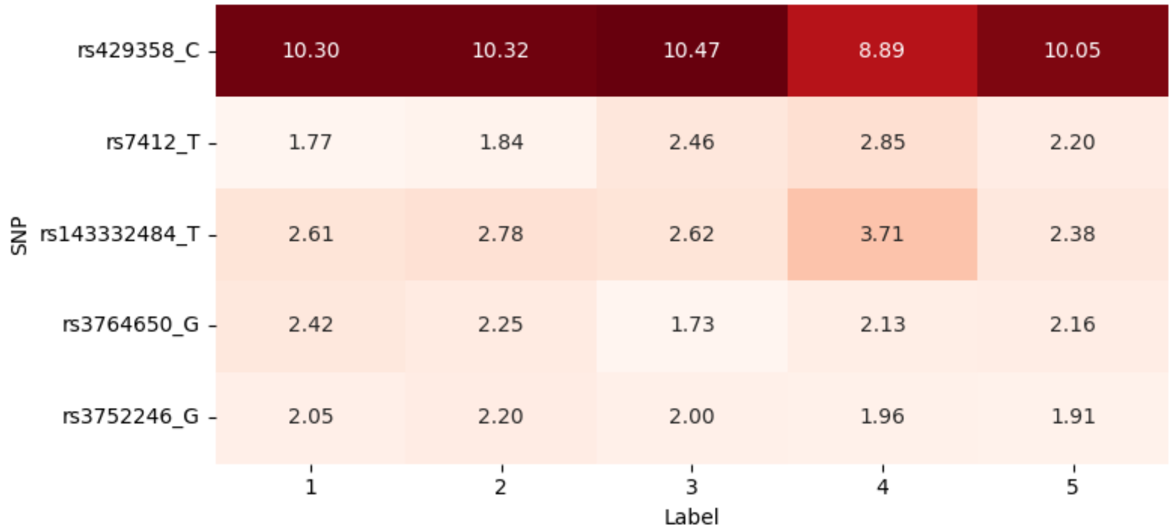

Supplementary Figure 25

Parkinson's disease (PD) carrier enrichment heatmap. This heatmap shows the carrier enrichment ratio for PD-associated single-nucleotide polymorphisms (SNPs) across the five PD clusters. The ratio was calculated as the cluster-specific carrier prevalence divided by the corresponding general population minor allele frequency.

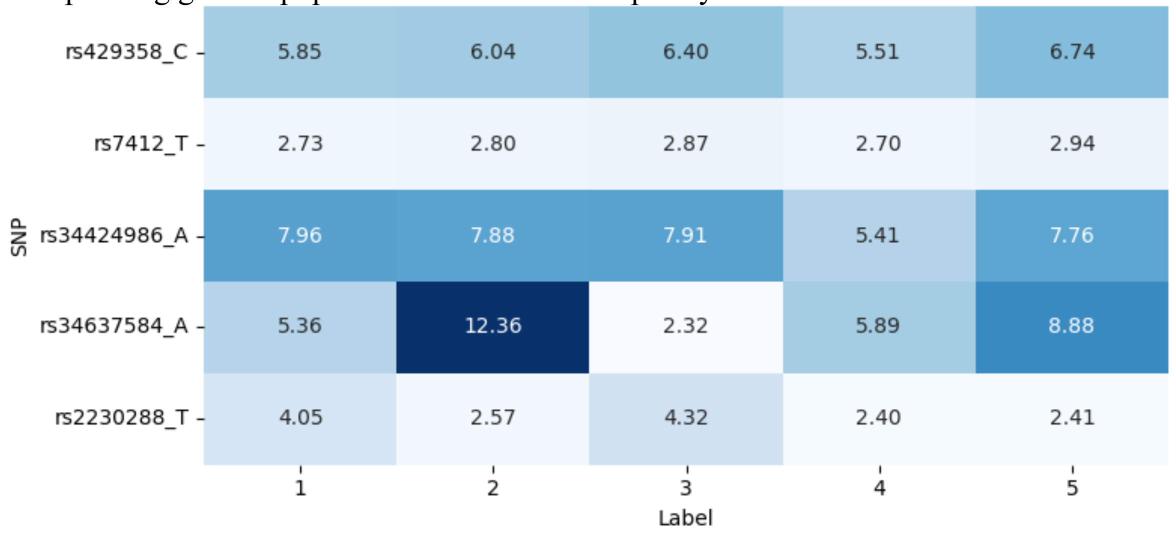

Supplement: Supplementary file 1 — Supplementary Methods 1–10 and Figs. 1–25. [file 43587_2026_1085_MOESM1_ESM.pdf]
