# Supplementary material for: Subtyping Alzheimer’s disease and Parkinson’s disease using longitudinal electronic health records
Source: Nat Aging. 2026 Feb 26;6(3):612–25. doi: 10.1038/s43587-026-01085-3 (PMC13004679; doi:10.1038/s43587-026-01085-3)
Supplement: Supplementary file 2 — Reporting Summary [file 43587_2026_1085_MOESM2_ESM.pdf]

nature portfolio

Corresponding author(s): Jie Lian, Kazem Rahimi

Last updated by author(s): Jan 16, 2026

Reporting Summary

Nature Portfolio wishes to improve the reproducibility of the work that we publish. This form provides structure for consistency and transparency in reporting. For further information on Nature Portfolio policies, see our Editorial Policies and the Editorial Policy Checklist.

Statistics

For all statistical analyses, confirm that the following items are present in the figure legend, table legend, main text, or Methods section.

n/a	Confirmed	
☐	☒	The exact sample size (*n*) for each experimental group/condition, given as a discrete number and unit of measurement
☐	☒	A statement on whether measurements were taken from distinct samples or whether the same sample was measured repeatedly
☐	☒	The statistical test(s) used AND whether they are one- or two-sided<br>*Only common tests should be described solely by name; describe more complex techniques in the Methods section.*
☐	☒	A description of all covariates tested
☐	☒	A description of any assumptions or corrections, such as tests of normality and adjustment for multiple comparisons
☐	☒	A full description of the statistical parameters including central tendency (e.g. means) or other basic estimates (e.g. regression coefficient) AND variation (e.g. standard deviation) or associated estimates of uncertainty (e.g. confidence intervals)
☐	☒	For null hypothesis testing, the test statistic (e.g. *F*, *t*, *r*) with confidence intervals, effect sizes, degrees of freedom and *P* value noted<br>*Give P values as exact values whenever suitable.*
☒	☐	For Bayesian analysis, information on the choice of priors and Markov chain Monte Carlo settings
☒	☐	For hierarchical and complex designs, identification of the appropriate level for tests and full reporting of outcomes
☐	☒	Estimates of effect sizes (e.g. Cohen's *d*, Pearson's *r*), indicating how they were calculated

Our web collection on statistics for biologists contains articles on many of the points above.

Software and code

Policy information about availability of computer code

Data collection	The datasets are public and were already collected. No software was used.
Data analysis	All analyses were performed in Python (version 3.7+). Deep learning models were implemented using PyTorch (v1.8.1) together with the Hugging Face Transformers library (v4.10.2). Data preprocessing used pandas (v1.3.5), and clustering analyses (k-means, silhouette score, Davies–Bouldin index, ARI) used scikit-learn (v1.0.2). Visualisations were produced using matplotlib (v3.5.3) and seaborn (v0.12.2). Dimensionality reduction for cluster visualisation was conducted using MulticoreTSNE (v0.1). Additional utilities included Grad-CAM (pytorch-gradcam, v0.2.1), torchvision (v0.9.1), torchaudio (v0.8.1), and torchdiffeq (v0.2.5).<br>All codes for model training and analyses were in Python. The code used for model training and subtyping is publicly available at: https://github.com/SereneLian/Subtyping_EHR_AD_PD.

For manuscripts utilizing custom algorithms or software that are central to the research but not yet described in published literature, software must be made available to editors and reviewers. We strongly encourage code deposition in a community repository (e.g. GitHub). See the Nature Portfolio guidelines for submitting code & software for further information.

Data

Policy information about availability of data

All manuscripts must include a data availability statement. This statement should provide the following information, where applicable:

- Accession codes, unique identifiers, or web links for publicly available datasets
- A description of any restrictions on data availability
- For clinical datasets or third party data, please ensure that the statement adheres to our policy

Access to Clinical Practice Research Datalink (CPRD) data, including UK primary care records and linked datasets such as Hospital Episode Statistics, is subject to approval through CPRD's Research Data Governance Process (https://www.cprd.com/research-applications). The UK Biobank data used in this study were obtained under approved application number 83942 and 116292, and are available to qualified researchers through UK Biobank's data access procedures.

Research involving human participants, their data, or biological material

Policy information about studies with human participants or human data. See also policy information about sex, gender (identity/presentation), and sexual orientation and race, ethnicity and racism.

Reporting on sex and gender	For CPRD Aurum, sex was taken from the demographic fields recorded by participants' general practices. In UK Biobank, sex was obtained from the self-reported baseline questionnaire. For all genetic analyses, we excluded individuals whose self-reported sex was discordant with their genetically inferred sex.
Reporting on race, ethnicity, or other socially relevant groupings	For CPRD Aurum, ethnicity, was taken from the demographic fields recorded by participants' general practices. In UK Biobank, ethnicity was obtained from the self-reported baseline questionnaire. For all genetic analyses, we excluded individuals whose self-reported ethnicity was discordant with their genetically inferred ethnicity
Population characteristics	There were 228,637 and 4,623 AD cases identified in CPRD and UK Biobank, from which 113,545 and 3,710 patients met our inclusion criteria. For PD, CPRD and UKB contained 95,408 and 4,685 cases respectively, with 45,825 and 3,732 patients respectively ultimately selected. The AD cohort's mean ages at diagnosis were 82.1 years (SD 8.0) in CPRD and 74.4 years (SD 5.5) in UK Biobank. Females comprised 63.8% of the CPRD cohort and 52.1% of the UK Biobank cohort. The cohorts were predominantly White (93.6% CPRD, 91.3% UK Biobank), with 22.3% (CPRD) and 34.5% (UK Biobank) classified as Index of Multiple Deprivation (IMD) category 1. Notably, 0.2% of AD patients in CPRD and 0.1% in UK Biobank were aged between 40 and 50 years. Additionally, 64.9% of CPRD patients were over 80 years old, compared to only 14.9% in UK Biobank.<br>In the PD cohorts, the mean ages at diagnosis were 77.8 years (SD 9.3) in CPRD and 70.6 years (SD 7.2) in UK Biobank, with females constituting 40.6% and 37.1% of each cohort, respectively. White individuals represented 93.3% of the CPRD and 90.70% of the UK Biobank cohorts. IMD category 1 was reported for 23.78% of CPRD and 37.88% of UK Biobank participants. Among PD patients aged 40-50 years, 0.93% were recorded in CPRD and 0.96% in UK Biobank. Additionally, 45.11% of CPRD patients were older than 80 years, in contrast to only 5.84% in the UK Biobank cohort.
Recruitment	CPRD, we selected patients who were aged 40 years and older, with incident reports of AD or PD between Jan 1, 2005 and Jan 1, 2018. UK Biobank patient were recruited between 2006-2010.
Ethics oversight	Access to UK Biobank data (application IDs 83942 and 116292) was obtained via UK Biobank's standard access procedures. UK Biobank holds blanket ethical approval from the North West Multicentre Research Ethics Committee to function as a research tissue bank; therefore, investigators who work under an approved application are covered by that approval and do not require separate ethics clearance.<br>The Clinical Practice Research Datalink (CPRD) has generic ethical approval from a National Research Ethics Service committee for all purely observational studies. Additional study-specific approval for this analysis was granted by the CPRD Independent Scientific Advisory Committee (protocol 20_095).

Note that full information on the approval of the study protocol must also be provided in the manuscript.

Field-specific reporting

Please select the one below that is the best fit for your research. If you are not sure, read the appropriate sections before making your selection.

☒ Life sciences ☐ Behavioural & social sciences ☐ Ecological, evolutionary & environmental sciences

For a reference copy of the document with all sections, see nature.com/documents/nr-reporting-summary-flat.pdf

Life sciences study design

All studies must disclose on these points even when the disclosure is negative.

Sample size	There were 228,637 and 4,623 AD cases identified in CPRD and UK Biobank, from which 113,545 and 3,710 patients met our inclusion criteria. For PD, CPRD and UKB contained 95,408 and 4,685 cases respectively, with 45,825 and 3,732 patients respectively ultimately selected.
Data exclusions	We selected patients who were aged 40 years and older, with incident reports of AD or PD between Jan 1, 2005 and Jan 1, 2018. Selection criteria included adherence to CPRD quality standards, eligibility for CPRD and HES linkage, and a minimum of 12 months of registration with

their GPs. The study period was restricted to 2005–2018 to maximise coding consistency and linkage completeness. Data quality in CPRD improved substantially after 2005 following the national implementation of SNOMED-CT and comprehensive HES linkage. The year 2018 was selected to ensure sufficient follow-up for post-diagnosis analyses.
AD and PD were identified based on the first recorded diagnostic code from linked primary care (GP records) or secondary care (HES) data. The date of this first code was defined as the index date of diagnosis. In primary care, we extracted diagnoses using Read or SNOMED CT code. In secondary care, we used ICD-10 codes. AD was identified using previously validated code lists (Supplementary Method 5), while PD was identified using ICD-10 code of G20. AD and PD cohorts were constructed separately. Patients carrying both codes were therefore included in both cohorts, as the AD and PD subtyping analyses were conducted independently.

Replication

We verified reproducibility through multiple complementary approaches. Five-fold cross-validation was performed within the derivation cohort to ensure stable and generalisable patient representations and subtype assignments. Model-derived subtypes were then replicated in an internal validation cohort and independently reproduced in an external dataset (UK Biobank). All replication attempts were successful, with consistent cluster structure and subtype characteristics across cohorts.

Randomization

Randomization was not applicable, as this was an observational study using routinely collected EHR data.

Blinding

Data collection and analysis were not performed blind to clinical outcomes, as all data were obtained from existing medical records.

Reporting for specific materials, systems and methods

We require information from authors about some types of materials, experimental systems and methods used in many studies. Here, indicate whether each material, system or method listed is relevant to your study. If you are not sure if a list item applies to your research, read the appropriate section before selecting a response.

Materials & experimental systems

n/a	Involved in the study
☒	☐ Antibodies
☒	☐ Eukaryotic cell lines
☒	☐ Palaeontology and archaeology
☒	☐ Animals and other organisms
☒	☐ Clinical data
☒	☐ Dual use research of concern
☒	☐ Plants

Methods

n/a	Involved in the study
☒	☐ ChIP-seq
☒	☐ Flow cytometry
☒	☐ MRI-based neuroimaging

Plants

Seed stocks

Report on the source of all seed stocks or other plant material used. If applicable, state the seed stock centre and catalogue number. If plant specimens were collected from the field, describe the collection location, date and sampling procedures.

Novel plant genotypes

Describe the methods by which all novel plant genotypes were produced. This includes those generated by transgenic approaches, gene editing, chemical/radiation-based mutagenesis and hybridization. For transgenic lines, describe the transformation method, the number of independent lines analyzed and the generation upon which experiments were performed. For gene-edited lines, describe the editor used, the endogenous sequence targeted for editing, the targeting guide RNA sequence (if applicable) and how the editor was applied.

Authentication

Describe any authentication procedures for each seed stock used or novel genotype generated. Describe any experiments used to assess the effect of a mutation and, where applicable, how potential secondary effects (e.g. second site T-DNA insertions, mosiacism, off-target gene editing) were examined.
